# Interfacial Coupling Design Enhancing Hole Transport in PTAA-Based Perovskite Solar Cells with Efficiency over 26%

**DOI:** 10.1007/s40820-026-02145-4

**Published:** 2026-03-18

**Authors:** Huaiman Cao, Xufan Zheng, Yue Qiang, Liangyu Zhao, Yulong Chen, Zhiguang Sun, Yingguo Yang, Hin-Lap Yip, Ze Yu

**Affiliations:** 1 State Key Laboratory of Fine Chemicals, Frontiers Science Center for Smart Materials Oriented Chemical Engineering, School of Chemical Engineering, Dalian University of Technology, Dalian, 116024 People’s Republic of China; 2 Department of Materials Science and Engineering, City University of Hong Kong, Kowloon, , 999077 Hong Kong People’s Republic of China; 3 Instrumental Analysis Center, Dalian University of Technology, Dalian, 116024 People’s Republic of China; 4 School of Microelectronics, Fudan University, Shanghai, 200433 People’s Republic of China; 5 Shanghai Synchrotron Radiation Facility (SSRF), Shanghai Institute of Applied Physics, Shanghai Advanced Research Institute, Chinese Academy of Sciences, Zhangjiang Lab, Shanghai, 201800 People’s Republic of China; 6 School of Energy and Environment, City University of Hong Kong, Kowloon, 999077 Hong Kong People’s Republic of China; 7 State Key Laboratory of Marine Environmental Health, Hong Kong Institute for Clean Energy, City University of Hong Kong, Kowloon, 999077 Hong Kong People’s Republic of China

**Keywords:** 2D/3D perovskites, Perovskite solar cells, *π*-Conjugated ligands, PTAA, High efficiency

## Abstract

Constructing 2D/3D perovskite heterojunction is an effective method to improve performance and stability of perovskite solar cells (PSCs), while the quantum well in 2D perovskites hinders carrier transport. To address this issue, *π*-conjugated semiconducting ligands have been introduced to enhance carrier-transfer capability of 2D perovskites. Here, two triphenylamine (TPA)-based ligands are specifically designed through *π*-extension with a fused (N-TPEAI) or covalently linked (P-TPEAI) benzene ring. For the first time, TPA semiconductor-based ligands have been incorporated to construct 2D/3D PSCs with poly[bis(4-phenyl)(2,4,6-trimethylphenyl)amine] (PTAA) as hole-transport materials (HTMs). Combined experimental and computational analyses reveal that this *π*-conjugation extension strategy proves to be effective in strengthening intermolecular interactions both between the adjacent spacer cations within 2D perovskites and at perovskite/PTAA interfaces, particularly in the case of P-TPEAI. Ultimately, the resultant 2D/3D PSCs employing P-TPEAI achieve an outstanding efficiency of 26.13%, which, to the best of our knowledge, is the highest value reported for 2D/3D PSCs incorporating PTAA HTMs. Moreover, benefiting from the robustness of both 2D perovskites and PTAA, the corresponding devices also exhibit excellent light-heat stability, meeting ISOS-L-2 protocol. These findings provide important guidelines for future design of organic spacers in advancing efficient and robust PSCs and related optoelectronic devices.

**Supplementary Information:**

The online version contains supplementary material available at 10.1007/s40820-026-02145-4.

## Highlights

- Two triphenylamine-based semiconducting ligands, namely N-TPEAI and P-TPEAI, were designed by extending the π-conjugation, with structural similarities to the backbone of poly[bis(4-phenyl)(2,4,6-trimethylphenyl)amine] (PTAA).
- This* π*-conjugation extension strategy proves to be effective in strengthening intermolecular interactions both between adjacent spacer cations within 2D perovskites and at perovskite/PTAA interfaces.
- The resultant 2D/3D perovskite solar cells (PSCs) employing P-TPEAI achieve an outstanding efficiency of 26.13%, which is the highest value reported for 2D/3D PSCs incorporating PTAA hole-transport layers.

**Supplementary Information:**

The online version contains supplementary material available at 10.1007/s40820-026-02145-4.

## Graphical abstract

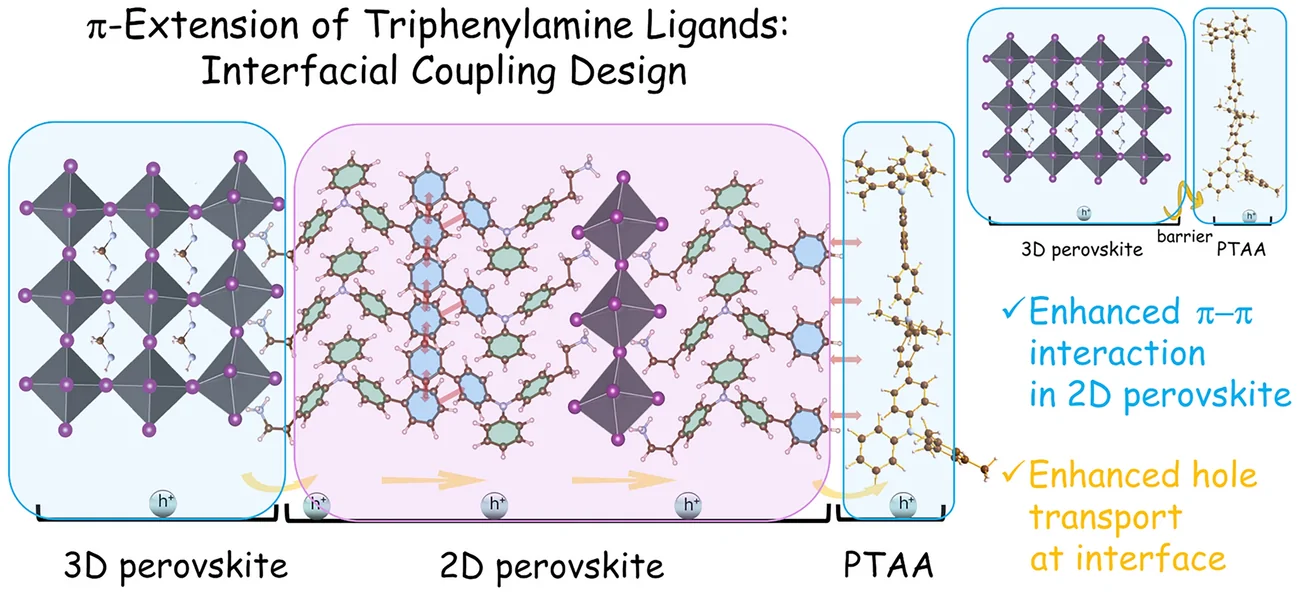

## Introduction

Over the past decade, the photovoltaic community has witnessed skyrocketed performance enhancement of perovskite solar cells (PSCs), benefiting from compositional engineering, defect passivation, and charge-selective layer optimization [1–5]. However, devices suffer from inevitable interfacial and bulky defects, resulting in serious performance degradation and unacceptable thermal and optional stability [2, 6]. Constructing 2D/3D perovskite heterojunctions has been demonstrated to be an effective approach in enhancing both the performance and stability of PSCs [7–12]. To overcome the inherent carrier-transport issue in 2D/3D perovskite heterojunction [13, 14], larger *π*-conjugated ligands (with at least two connected aromatic groups) have garnered considerable interest from the researchers [7, 13, 15–19].

*π*-conjugated ligands in 2D perovskites are mainly divided into the following categories: alkyl ammonium salts with the structure of (1) multithiophene [20, 21] or fused thiophene [22]; (2) biphenyl [23], naphthacene [3, 24], anthracene [25], pyrene [7], and perylene [26]; (3) aromatic imides [17]; and (4) triphenylamine (TPA)-based ligands. A series of conjugated quaterthiophene ligands have been applied to form 2D/3D perovskite heterojunction in poly[bis(4-phenyl)(2,4,6-trimethylphenyl)amine] (PTAA)-based devices, which resulted in the improvements of both energy-level alignment at 2D/3D perovskite heterojunction and the perovskite/PTAA interface quality due to the *π*-conjugated effect [15, 21]. For ligands with large *π*-conjugated structures, they have been used by many research groups to balance the passivation effect and carrier transport in 2D perovskites [7, 16]. However, there have been only few works focusing on TPA-based ligands, although TPA moiety is a well-known electron donor and has been widely used in optoelectronic devices, including dye-sensitized solar cells, organic solar cells, and light-emitting diode [27]. Qin and co-workers reported an efficient bifunctional organic salt (TA-PMA), consisting of hole-transporting unit (TPA) and 2D-dimensionalizing unit (phenylmethylammonium, PMA). The formation of hole-transporting 2D peroskite on top of 3D perovskites not only significantly reduces interface trap density but also enhances hole-extracting abilities of 2D/3D heterojunction region [28]. Recently, our group reported a new TPA-based hole-transporting ligand (DPA-PEAI), the propeller-like geometry of which enables the formation of multifarious *π*−*π* interactions within (DPA-PEA)_2_PbI_4_ 2D perovskite. As a result, 2D/3D FAPbI_3_-based PSCs employing DPA-PEAI afford a decent efficiency of 25.7% [29]. Nevertheless, TPA-based ligands are still far from fully explored, given their structural tunability and the potential to further optimize the performance of PSCs.

**Fig. 1:**
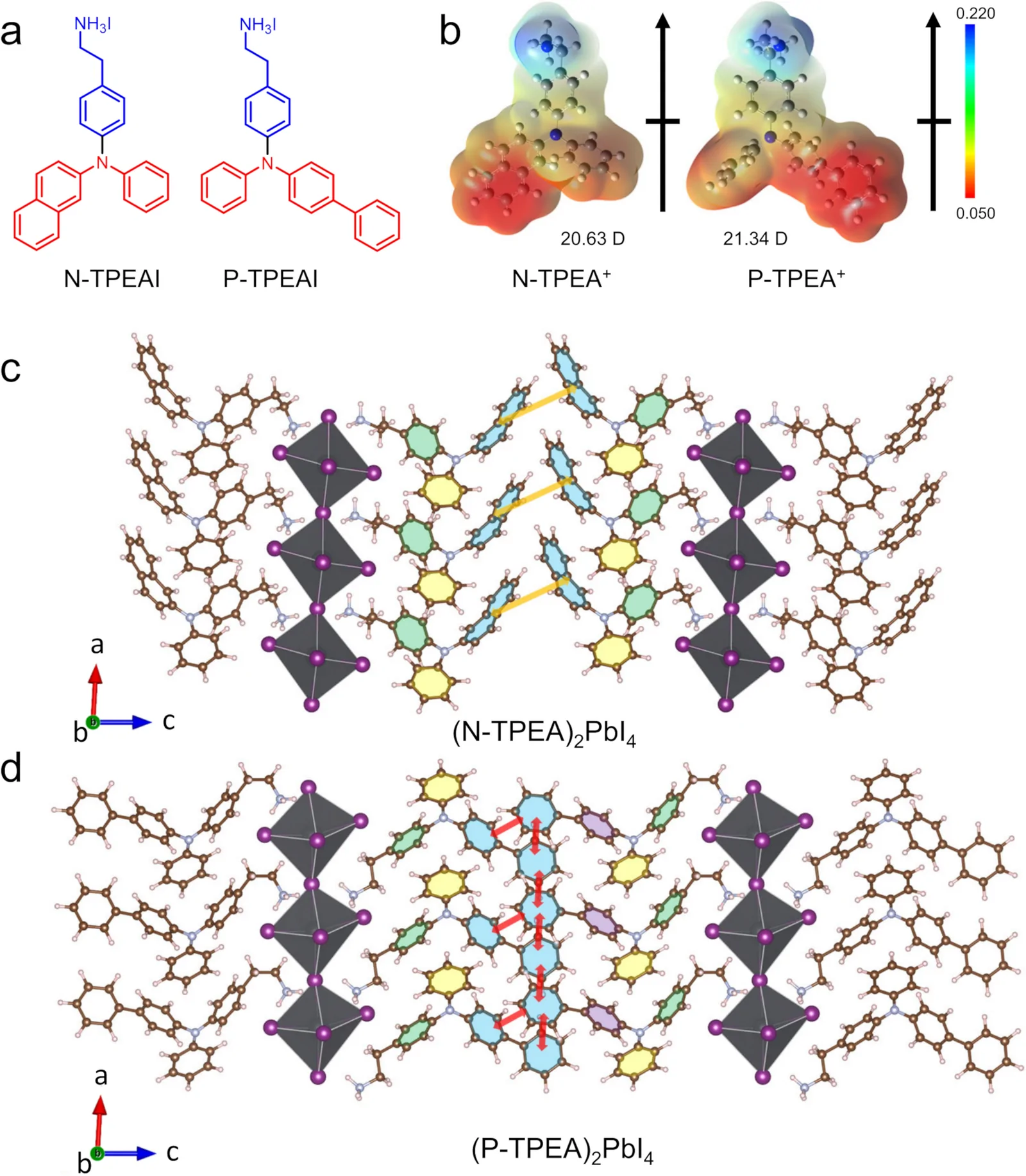
**a** Chemical structures of N-TPEAI and P-TPEAI.** b** Electrostatic surface potential (ESP) profiles of N-TPEA^+^ and P-TPEA^+^ cations. Simulated 2D perovskite crystal structures of** c** (N-TPEA)_2_PbI_4_ and **d** (P-TPEA)_2_PbI_4_

Here, we designed and synthesized two novel TPA-based semiconducting ligands by extending *π*-conjugation, namely N-TPEAI and P-TPEAI, following our previous work (Fig. 1a). The only difference between N-TPEAI and P-TPEAI lies in the attachment pattern of another benzene ring to one of the phenyls of diphenyl moiety in DPA-PEAI: fusing in N-TPEAI and covalently linking in P-TPEAI. Our motivations are two-fold: first, we aimed to gain a deep understanding of the influence of this structural change in the ligands on the property of 2D perovskites; second, we also intended to study the intermolecular interactions between the ligands and PTAA, due to their structural similarities to the backbone of PTAA. Combined density functional theory (DFT) simulation and experimental results demonstrated that the *π*-extension mode in P-TPEAI had more pronounced impacts on strengthening the intermolecular interactions both between the adjacent spacer cations within 2D perovskites and at perovskite/PTAA interfaces, leading to synergistic hole-transfer promotion in the devices. As a consequence, PSCs with P-TPEAI deliver an excellent power conversion efficiency (PCE) of 26.13%, which is significantly higher than those of the devices using N-TPEAI (25.05%) and without modifications (21.69%).

## Experimental Sections

Further details regarding materials, synthesis, device fabrication, characterizations, and calculational method, as well as supporting figures and tables, are available in the Supplementary material.

## Results and Discussion

### Molecular Design and Simulated 2D Perovskite Structures

N-TPEAI and P-TPEAI were synthesized as reported previously [29], and the details of the synthetic procedures are depicted in the Supporting Information (Scheme S1 and Figs. S1 − S6). DFT calculation was first performed to evaluate the electrostatic surface potential (ESP) profiles and dipole moments of N-TPEA^+^ and P-TPEA^+^ cations, as shown in Fig. 1b. A larger dipole moment of 21.34 Debye is identified for P-TPEA^+^ as compared to that of N-TPEA^+^ (20.63 Debye), implying that more effective charge separation in 2D perovskites can be expected in the former case, associated with favourable hole extraction process [30].

Next, we prepared *n* = 1 2D perovskites by spin-coating the solution of lead iodide (PbI_2_) with N-TPEAI or P-TPEAI in dimethylformamide (DMF) on fluorine-doped tin oxide (FTO) substrates. Both films show the characteristic peak of *X*-ray diffraction (XRD) patterns at 3.14° and 3.28° in the low-angle region, respectively, which should be assigned to the 2D perovskites (P-TPEA)_2_PbI_4_ and (N-TPEA)_2_PbI_4_ (Fig. S7). The corresponding lattice spacing for (P-TPEA)_2_PbI_4_ is estimated to be 28.13 Å, which is larger than that of (N-TPEA)_2_PbI_4_ (26.93 Å), owing to the relatively bulky size of P-TPEA^+^ (472 Å^3^) with respect to N-TPEA^+^ (436 Å^3^). Unfortunately, we encountered challenges to obtain high-quality single crystals of 2D perovskites, presumably because of the high formation energy [31], which will be discussed in the following section. Instead, we conducted DFT calculations to simulate the structures of 2D perovskites to gain insights into the preferable packing arrangements of the ligands. The simulations were performed by using the generalized gradient approximation (GGA) of the Perdew–Burke–Ernzerhof (PBE) exchange–correlation functional and the projector augmented-wave (PAW) pseudopotential method [7, 32, 33]. The details of DFT calculations are described in Supporting Information, and the results are illustrated in Fig. 1c, d and Table S1. The lattice spacing of (N-TPEA)_2_PbI_4_ and (P-TPEA)_2_PbI_4_ is calculated to be 27.01 and 29.10 Å, respectively, in good agreement with the corresponding values obtained from the XRD measurements.

Interestingly, the *π*-extension mode in the ligands was found to have a profound impact on their packing configurations in the organic layers of 2D perovskites. As presented in Fig. 1c, for (N-TPEA)_2_PbI_4_, edge-to-face stacking is observed between the naphthalene moieties in the tail part of N-TPEA^+^ [34], probably due to the larger steric hindrance of naphthalene. The corresponding ring centroid-to-centroid distances (*R*_cc_) and the angle of two naphthalene rings are estimated to be 5.69 Å and 86.5°, respectively (Fig. S8 and Table S2). This arrangement of naphthalene units allows the formation of herringbone-like stacking geometry between the adjacent organic layers of (N-TPEA)_2_PbI_4_ [35]. By stark contrast, the biphenyl units, the tails of P-TPEA^+^, prefer to stack with parallel-slipped mode (Fig. 1d), most likely because the covalently linked phenyl group can rotate freely. The peripheral benzene ring of P-TPEA^+^ tends to construct *π*-*π* orbital overlap with both of the two benzene rings of the biphenyl moiety in the neighbouring cation from another organic layer. The corresponding dihedral angles of phenyl planes are calculated to be 27.7° and 27.9°, respectively, with smaller *R*_cc_ of 4.27 and 4.30 Å (Fig. S9 and Table S3) [36]. This crossing parallel-displaced packing of the phenyl groups will provide multifarious channels for charge transport, which is beneficial to the out-of-plane hole transport in (P-TPEA)_2_PbI_4_. Moreover, the average of in-plane Pb–I–Pb bond angles are estimated to be 169.8° and 167.0° (along axles* a* and* b*), with the average Pb–I bond length of 3.15 and 3.16 Å (horizontal) in (N-TPEA)_2_PbI_4_ and (P-TPEA)_2_PbI_4_, respectively (Table S1). The relatively larger Pb–I–Pb bond angles observed indicate smaller deformation of [PbI_6_]^4−^ inorganic layer, associated with better stability and carrier mobility in both 2D perovskites [14, 34, 37]. Additionally, the formation energy is calculated to be −2.96 eV for (P-TPEA)_2_PbI_4_, which is larger than that of −3.38 eV for (N-TPEA)_2_PbI_4_. It has been reported that 2D perovskites with higher formation energies show weakened cation exchange process, supressed ion migration, and better long-term stability [31].

The binding energy (*E*_b_) and hole-transfer integral (HTI) values were further calculated to quantitatively evaluate the intermolecular interactions between two pairs of the adjacent spacer cations (Tables S2 and S3). Larger *E*_b_ values (−16.42 and −16.15 eV) are identified for P-TPEA^+^ cation pairs in comparison to those of N-TPEA^+^ counterparts (−15.80 and −15.68 eV), indicating stronger intermolecular interactions in the former case. This result correlates well with more *π *− *π* orbital overlap observed in the simulated structures for (P-TPEA)_2_PbI_4_ 2D perovskites. On the other hand, the HTI values for both two systems are considerably larger (P-TPEA^+^: 118.8 and 108.7 meV; N-TPEA^+^: 131.8 and 50.5 meV), relative to the corresponding values for DPA-PEAI in the previous report [29]. This comparison highlights that both the two *π*-extension modes are effective in enhancing the hole-transfer property within 2D perovskites. In the case for N-TPEA^+^ cation, the observed improved hole-transfer capability should be most likely attributed to the relatively larger *π*-conjugation of the naphthalene [7, 16], although *π*-orbital overlap is not pronounced in the edge-to-face stacking motifs [34].

Furthermore, larger *E*_b_ and HFI values are found between P-TPEA^+^ cation and PTAA as compared to those for N-TPEA^+^/PTAA and DPA-PEA^+^/PTAA counterpart (Fig. S10 and Table S4), most likely due to more *π*−*π* orbital overlap of benzenes in the former pair associated with smaller rigidity of biphenyl unit [29, 38]. Overall, the combined DFT calculation results indicate that the *π*-conjugation expansion of the ligands is beneficial for the hole-transport processes not only within 2D perovskites but also at the interface of perovskites and PTAA, especially in the case of P-TPEAI.

### Photophysical Properties of 2D/3D Perovskite Films

**Fig. 2:**
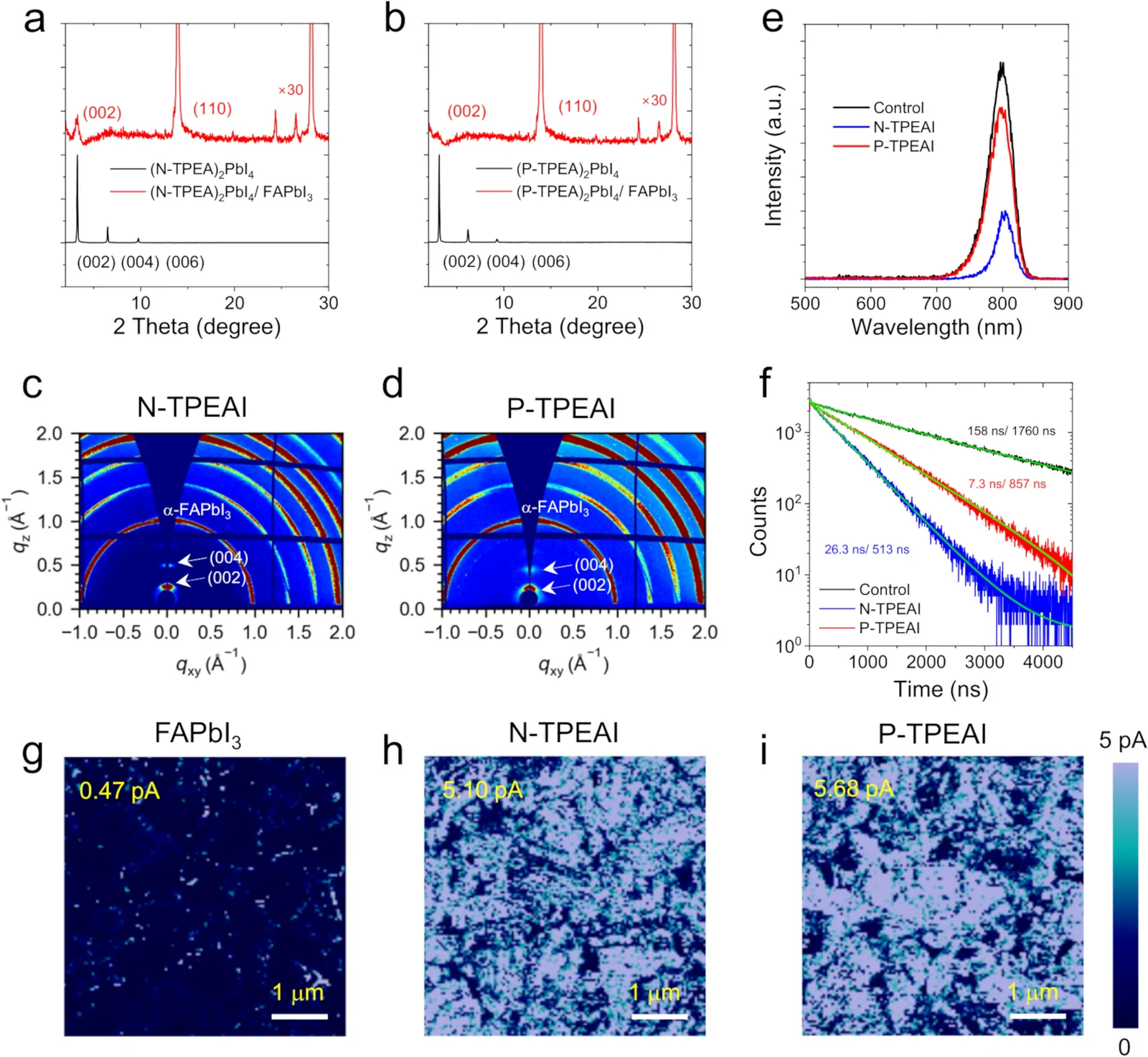
XRD patterns of **a** (N-TPEA)_2_PbI_4_ and N-TPEAI-modified FAPbI_3_ films; **b** (P-TPEA)_2_PbI_4_ and P-TPEAI-modified FAPbI_3_ films. 2D GIWAXS patterns of **c** N-TPEAI- and **d** P-TPEAI-modified FAPbI_3_ films on silica wafer substrates. **e** Steady-state PL and **f** TRPL kinetics of pristine and treated perovskite films. *C*-AFM images of **g** FAPbI_3_, **h** N-TPEAI-treated, and **i** P-TPEAI-treated perovskite films

2D/3D perovskite heterojunctions were further prepared by spin-coating the solution of N-TPEAI and P-TPEAI in chloroform (CF) on the surface of FAPbI_3_, followed by thermal annealing at 120 °C [39]. XRD results show that both the treated films present respective diffraction peak of low-dimensional perovskites (2D) (Figs. 2a, b and S11), in good agreement with the pure (N-TPEA)_2_PbI_4_ and (P-TPEA)_2_PbI_4_. Grazing incidence wide-angle *X*-ray scattering (GIWAXS) characterizations at incidence angle of 0.30° further confirm the formation of out-of-plane prefer-orientation 2D perovskites at the surface of 3D perovskites. Distinct signals at *q*_z_ = ~ 0.24 Å^−1^ (002) and* q*_z_ = ~ 0.48 Å^−1^ (004) are detected for N-TPEAI-modified films, while signals at *q*_z_ = ~ 0.22 Å^−1^ (002) and* q*_z_ = ~ 0.44 Å^−1^ (004) are observed for P-TPEAI-treated films, which could be attributed to corresponding 2D perovskites, respectively (Figs. 2c, d and S12). The P-TPEAI-treated film shows a high degree of vertically orientated perovskite phases and lower crystallinity.

Next, we performed a set of experiments to evaluate the charge-transport property of perovskite films with and without modifications. Steady-state photoluminescence (PL) results show that PL quenching is observed for both the treated films (Fig. 2e), suggesting the direct hole extraction from 3D perovskites to 2D perovskites, strongly correlated with the semiconducting characteristics of these two ligands [15, 20, 21, 40]. The PL mapping also shows that the treated samples exhibit significantly more homogeneous PL intensity distribution and lower intensity compared to the control film (Fig. S13). We subsequently conducted time-resolved PL (TRPL) measurements to gain insights into the charge-transfer dynamics of the perovskite films (Fig. 2f), and the TRPL kinetics were fitted with a biexponential decay function (Table S5). At the initial stage, both the treated films exhibit faster PL decay (7.3 ns for P-TPEAI and 26 ns for N-TPEAI) in comparison with that of the control film (158 ns), indicating faster charge extraction process occurring in the treated films, in particular for P-TPEAI. The partial enlarged details of TRPL spectra at the initial stage are also presented in Fig. S14. Additionally, similar trend of TRPL decay at initial stage is also observed for the perovskite films coated with PTAA (Fig. S15 and Table S5), with P-TPEAI-treated sample showing a faster hole extraction process occurs at perovskite/PTAA interfaces. Space-charge-limited current (SCLC) and conductive atomic force microscopy (*C*-AFM) characterizations were also performed to examine the electronic property of the perovskite films with and without treatments (Figs. 2g–i and S16). The samples with P-TPEAI treatment exhibit the highest hole mobility (4.55 × 10^−4^ cm^2^ V^−1^ S^−1^) and current signal value (5.68 pA) among the films tested. Taken together, the above experimental examinations indicate that the hole-transport capability is significantly enhanced both inside 2D perovskites and at perovskite/PTAA interfaces for P-TPEAI-treated films, in good consistency with DFT calculations.

We conducted scanning electron microscopy (SEM) and atomic force microscopy (AFM) to evaluate the surface morphology of the perovskite films before and after modifications (Figs. S17 and S18). Both the treated films illustrate smoother surface and lower surface roughness as compared to the control films, which is consistent with the PL mapping results. *X*-ray photoelectron spectroscopy (XPS) measurements were implemented to probe the surface states of the perovskite films (Fig. S19). In comparison with the pristine films, the Pb 4*f*_7/2_ and I 3*d*_3/2_ peaks are obviously shifted towards lower binding energies for both the treated films, in particular for the P-TPEAI-treated sample. These results indicate that the passivation effect of P-TPEAI on the surface of perovskite is more prominent. This hypothesis gained supports from the SCLC measurements (Fig. S16 and Table S6), where the P-TPEAI-treated films present the lowest trap-filled limit voltage (*V*_TFL_) and trap density (*N*_trap_).

### Energy-Level Arrangement of 2D/3D Perovskite Films

Cyclic voltammetry (CV) and UV-Vis absorption measurements were further carried out to evaluate the energy levels of the ligands (Figs. S20–S21 and Table S7). It is found that the type II energy-level arrangements between the inorganic sheets [PbI_6_]^4−^ and organic cations are both achieved within these two 2D perovskite structures (Fig. S22), which is helpful for the out-of-plane charge transport [15]. Notably, a narrower bandgap of (P-TPEA)_2_PbI_4_ is determined as compared to that of (N-TPEA)_2_PbI_4_, suggesting that better photoelectric properties can be expected in the former system [14].

**Fig. 3:**
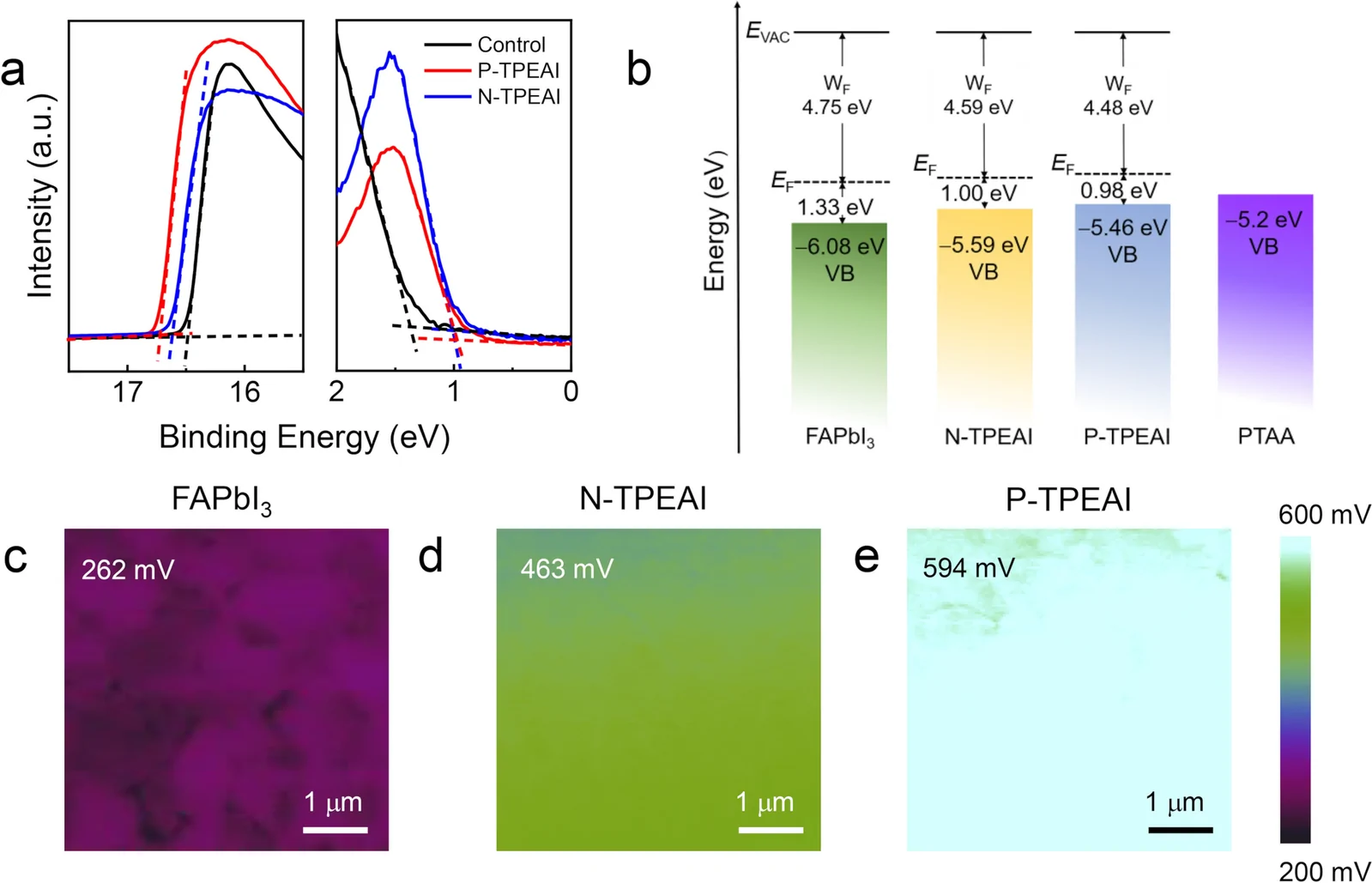
**a** Ultraviolet photoelectron spectroscopy (UPS) of secondary electron cut-off region (left) and the valance band region (right). **b** An energy-level diagram was derived from UPS before and after surface modification. KPFM images of **c** FAPbI_3_, **d** N-TPEAI-treated, and **e** P-TPEAI-treated perovskite films

We subsequently conducted ultraviolet photoelectron spectroscopy (UPS) measurements to examine the energy-level arrangements at 2D/3D perovskite heterojunction. Both treated films exhibit upshifted valence band maximum (VBM) and narrower gap between Fermi level (*E*_F_) and VBM, indicating more *p*-type surface for 2D/3D perovskites (Fig. 3a, b). Type II energy-level arrangements between perovskites and PTAA (with a HOMO of − 5.2 eV) are also observed for both cases (Fig. 3b) [41]. It is also noticeable that the film treated with P-TPEAI shows a smaller gap between the VBM of perovskite and HOMO of PTAA, which benefits the charge transport at interfaces. Kelvin probe force microscopy (KPFM) measurements (Fig. 3c–e) further revealed that both 2D/3D perovskite films display an increase in the mean surface potential relative to the control film, in consistency with the work function (*W*_F_) reduction observed for the perovskite films with treatments in UPS. The reduced hole injection barrier between perovskite film and PTAA will promote the hole-transfer process, in agreement with the DFT calculations.

### Device Characterizations and Stability

**Fig. 4:**
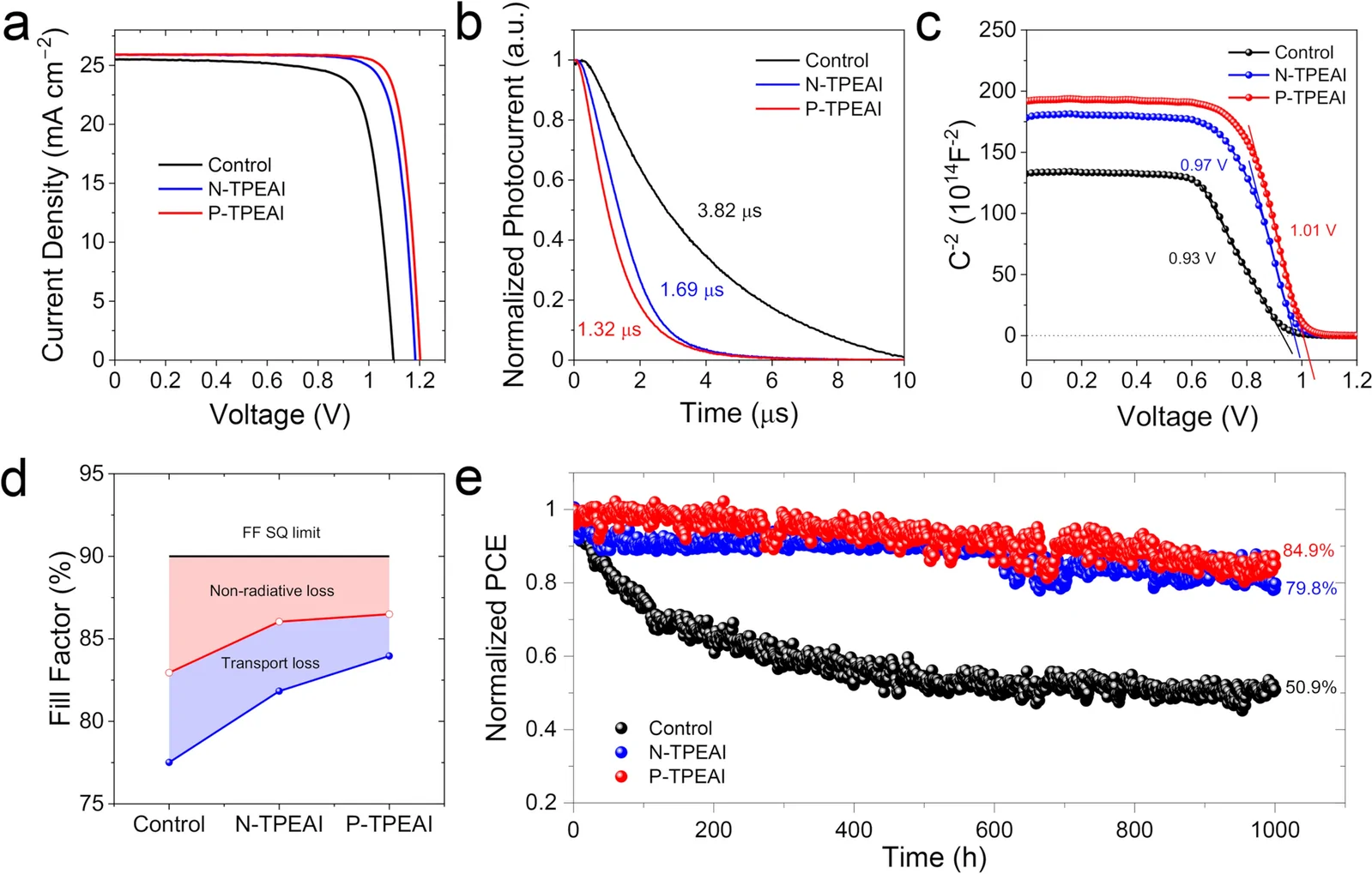
**a** *J*–*V* characteristics of the best-performing PSCs. **b** Transient photocurrent decay of PSCs with and without treatment. **c** Mott–Schottky fitting to the capacitance–voltage (*C*^−2^ − *V*) plots of different PSCs. **d** The device FF Shockley–Queisser (S–Q) limit consists of nonradiative loss (red region) and charge-transport loss (blue region). The solid and open circles stand for the measured FF and the maximum FF without charge-transport loss, respectively. **e** MPP tracking of unencapsulated PSCs under continuous one-sun illumination in an N_2_ environment at 65 °C. The initial PCE of control, N-TPEAI-treated, and P-TPEAI-treated devices is 19.1%, 23.5%, and 24.6%, respectively

To evaluate the efficacy of these two newly synthesized ligands, we constructed *n*-*i*-*p* PSCs with a planar architecture of FTO/SnO_2_/FAPbI_3_/surface treatment/PTAA/Au. The corresponding cross-sectional SEM image is presented in Fig. S23. The optimal concentrations of both ammonium salts were determined to be 3 mg mL^−1^ in CF (Tables S8 and S9). The current density–voltage (*J–V*) curves of the best-performing devices recorded under one-sun irradiation (100 mW cm^−2^, AM 1.5G) are displayed in Figs. 4a and S24, and the corresponding parameters are summarized in Table S10. The devices with P-TPEAI deliver an impressive PCE of 26.13%, with an open open-circuit voltage (*V*_oc_) of 1.201 V, a short-circuit current density (*J*_sc_) of 25.91 mA cm^−2^, and a fill factor (FF) of 83.96%. To our knowledge, this is the highest value reported for 2D/3D PSCs based on PTAA HTLs (Table S11). By comparison, N-TPEAI-treated devices also present an improved PCE (25.05%) relative to that of the control devices (21.69%) and DPA-PEAI-treated devices (24.96%) (Fig. S25). The devices with Spiro-OMeTAD as HTL were also prepared; PCEs of 24.96% and 24.59% were obtained for P-TPEAI- and N-TPEAI-treated devices, respectively (Fig. S26), which indicate the superiority of these ligands in application of PTAA-based devices, owing to the structural similarity between ligands and PTAA. Furthermore, PSCs incorporating P-TPEAI also exhibit the smallest hysteresis index, the highest stability power output (25.54%), and superior average PCE (25.64% ± 0.25%) (Figs. S27–S28 and Table S10). External quantum efficiency (EQE) measurements further verified that the integrated photocurrent densities are in good line with the *J–V* characterizations (Fig. S29).

Transient photocurrent (TPC) decay measurements were undertaken to gain insights into the charge-transfer dynamics for the PSCs with and without modifications, as illustrated in Fig. 4b. The P-TPEAI-treated devices present the shortest photocurrent decay lifetimes (1.32 μs), as compared to those of N-TPEAI-based (1.69 μs) and control (3.82 μs) devices. This result indicates that more efficient charge extraction and collection occur in the P-TPEAI-based devices, strongly correlated with the superior hole-transport capability both inside 2D perovskites and at perovskite/PTAA interfaces, as manifested by the computational and experimental analyses. Light intensity dependence of *V*_*oc*_ and Mott−Schottky measurements (Figs. 4c and S30) further demonstrated that P-TPEAI-treated PSCs exhibit the smallest ideality factor (*n*_id_) and largest built-in potential (*V*_bi_), associated with suppressed nonradiative recombination losses and improved carrier separation in these devices, which accounts for the observed enhancement of *V*_*oc*_. Transient photovoltage (TPV) decay measurements were also tested (Fig. S31), in which the longer photovoltage decay time indicates reduced defect density and suppressed nonradiative recombination for the treated devices. Moreover, we calculated FF losses in the devices from *J*–*V* curves and *n*_id_ values [42], and the corresponding results are illustrated in Fig. 4d. Both the nonradiative and charge-transport losses are identified to be reduced significantly in P-TPEAI-based PSCs, which well explains the much-improved FF obtained in such devices.

Finally, the long-term operational stability of unencapsulated PSCs was examined by tracking the maximum power point (MPP) under continuous one-sun illumination in a N_2_ environment at 65 °C, following the ISOS-L-2 protocol (Fig. 4e). The P-TPEAI-based PSCs showed superior photo-thermal stability, retaining ~ 85% of the initial performance after 1000 h, as compared to those of the devices without modification (~ 51%) and with N-TPEAI treatment (~ 80%). Besides, the water contact angles (Fig. S32) were measured to be 54.1°, 65.7°, and 72.5° for control, N-TPEAI- and P-TPEAI-treated perovskite films, respectively, indicating a better blocking effect of moisture for treated films. We also checked the photostability of the unencapsulated devices (Fig. S33) under continuous illumination at an open-circuit condition in ambient environment (~ 15 °C, 25%–30% RH). The control device decreased to 45% of its initial efficiency within 360 h. By contrast, the P-TPEAI- and N-TPEAI-treated devices maintain 89% and 80% of the initial performance after 504 h, respectively, demonstrating their much-improved photostability in air atmosphere.

## Conclusions

In summary, we developed two triphenylamine-based semiconducting ligands by extending the *π*-conjugation and applied them to fabricate 2D/3D PSCs in conjunction with PTAA HTLs. A systematic investigation, including DFT simulations and a set of experiments, revealed that the *π*-extension pattern, which is fused or covalently linked to another benzene ring, in the ligands, exerted a profound impact on the molecular packing arrangement between neighbouring spacer cations, and the intermolecular interactions between the ligands and PTAA. Ultimately, the hole-transfer capability was improved both inside the 2D perovskites and at perovskite/PTAA interfaces, in particular for the P-TPEAI ligands. This enhancement resulted in a remarkable PCE of 26.13% for PTAA-based PSCs, alongside outstanding light-heat durability. These encouraging outcomes highlight the importance of molecular engineering of *π*-conjugated ligands in promoting the performance of PSCs. By combining organic semiconductor-incorporated 2D perovskites and PTAA, we successfully established a system that integrated high efficiency and high light-heat-operational stability simultaneously. Furthermore, our molecular engineering approach offers various potential applications in other related devices, i.e. inverted or tandem PSCs, where charge-transport issue at perovskite/HTL interfaces also needs to be solved.

## Supplementary Information

Below is the link to the electronic supplementary material.

Supplementary file1 (DOCX 29800 KB)
