# Supplementary material for: Interfacial Coupling Design Enhancing Hole Transport in PTAA-Based Perovskite Solar Cells with Efficiency over 26%
Source: Nanomicro Lett. 2026 Mar 18;18:287. doi: 10.1007/s40820-026-02145-4 (PMC12996588; doi:10.1007/s40820-026-02145-4)
Supplement: Supplementary file 1 — Supplementary file1 (DOCX 29800 KB) [file 40820_2026_2145_MOESM1_ESM.docx]

*Supporting Information for*

**Interfacial Coupling Design Enhancing Hole Transport in PTAA-Based Perovskite Solar Cells with Efficiency over 26%**

Huaiman Cao^1, 2^, Xufan Zheng^1^, Yue Qiang^1^, Liangyu Zhao^1^, Yulong Chen^1^, Zhiguang Sun^3^, Yingguo Yang^4, 5,^ *, Hin-Lap Yip^2, 6, 7^ * and Ze Yu^1,^ *

^1^ State Key Laboratory of Fine Chemicals, Frontiers Science Center for Smart Materials Oriented Chemical Engineering, School of Chemical Engineering, Dalian University of Technology, Dalian 116024, P. R. China

^2^ Department of Materials Science and Engineering, City University of Hong Kong, Kowloon, Hong Kong 999077, P. R. China

^3^ Instrumental Analysis Center, Dalian University of Technology, Dalian 116024, P. R. China

^4^ School of Microelectronics, Fudan University, Shanghai 200433, P. R. China

^5^ Shanghai Synchrotron Radiation Facility (SSRF), Zhangjiang Lab, Shanghai Advanced Research Institute, Shanghai Institute of Applied Physics, Chinese Academy of Sciences, Shanghai 201800, P. R. China

^6^ School of Energy and Environment, City University of Hong Kong, Kowloon, Hong Kong 999077, P. R. China

^7^ Hong Kong Institute for Clean Energy, State Key Laboratory of Marine Environmental Health, City University of Hong Kong, Kowloon, Hong Kong 999077, P. R. China

*Corresponding author. E-mail: ze.yu@dlut.edu.cn (Z. Yu); a.yip@cityu.edu.hk (H-L. Yip); yangyingguo@fudan.edu.cn (Y. Yang)

This file contains:

Materials, synthesis, device fabrication, characterizations, and calculational method.

Supplementary Figure S1−S33

Supplementary Table S1−S11

Supplementary References

1. Materials

Lead iodide (PbI_2_, 99%) and Lead brommide (PbBr_2_, 98%) were purchased from Tokyo Chemical Industry. Formamidinium iodide (FAI, 99.9%), methylammonium chloride (MACl, 99.9%), and methylammonium bromide (MABr, 99.9%) were obtained from Advanced Election Technology. Poly[bis(4-phenyl)(2,4,6-trimethylphenyl)amine] (PTAA, *M*_n_ = 15.000−25.000) was purchased from Xi’an Polymer Light Technology Corporation. Tin (II) chloride dihydrate (SnCl_2_·2H_2_O, 99.99%) and thioglycolic acid (C_2_H_4_O_2_S) were obtained from Sigma-Aldrich. Urea (CO(NH_2_)_2_), potassium chloride (KCl), hydroiodic acid (HI, ca. 55 wt%), tris(dibenzylideneacetone)dipalladium (Pd_2_(dpa)_3_), 4-isopropyl-4’-methyldiphenyliodonium tetrakis(pentafluorophenyl)borate (TPFB), tri-*tert*-butylphosphine tetrafluoroborate (*tt-*BP·HBF_4_), *N*-Phenyl-4-biphenylamine (P-DPA), *N*-Phenyl-2-naphthylamine (N-DPA), trifluoroacetic acid (TFA), 2-(4-bromophenyl)ethylamine (4-Br-PEA), and di-*tert*-butyl decarbonate were purchased from Energy Chemical. Anhydrous chlorobenzene (CB), dimethyl sulfoxide (DMSO), and *N*,*N*-dimethylformamide (DMF) were obtained from J&K Scientific.

2. Synthesis

**Scheme S1.** Synthetic route for N-TPEAI and P-TPEAI.

(a) DCM, di-*tert*-butyl decarbonate, ice bath, RT, 18 h, yield: 96%; (b) *N*-phenyl-2-naphthylamine, Pd_2_(dba)_3_, tri-*tert*-butylphosphine tetrafluoroborate, sodium *tert*-butoxide, dry toluene, 120 °C, 18 h, yield: 57%; (c) *N*-phenyl-4-biphenylamine, Pd_2_(dba)_3_, tri-*tert*-butylphosphine tetrafluoroborate, sodium *tert*-butoxide, dry toluene, 120 °C, 18 h, yield: 66%; (d) Ethyl acetate, HI, ice bath, 1 h, RT, 2 h, yield: 70%; (e) Ethyl acetate, HI, ice bath, 1 h, RT, 2 h, yield: 72%.

The synthetic routes for N-TPEAI and N-TPEAI are depicted in Scheme S1. Compound **1** was prepared according to a previous report [1].

*Synthesis of N-TPEA-Boc* [2]

*N*-Phenyl-2-naphthylamine (722.7 mg, 3.30 mmol), tris-(dibenzylideneacetone)dipalladium(0) (90 mg), compound **1** (1018 mg, 3.40 mmol), sodium *tert*-butoxide (220 mg), and *tri*-*tert*-butylphosphine tetrafluoroborate (26 mg) were dissolved into anhydrous toluene (5 mL) in a 50 mL two-necked flask. The reaction was heated to 120 °C for 18 h. After cooling to RT, the mixture was extracted with brine water and dichloromethane. The organic phase was collected and the solution was evaporated under reduced pressure. The crude product was purified by column chromatography (silica gel, ethyl acetate (EA)/ PE = 1:10, v/v) to give a white solid (823 mg, 57%).  ^1^H NMR (DMSO-*d*6, 400 MHz): *δ* 7.83 (d, 2H, *J* = 8.58 Hz), 7.68 (d, 1H, *J* = 7.80 Hz), 7.43−7.35 (m, 3H, *J* = 9.37 Hz), 7.31 (dd, 2H, *J* = 8.19 Hz), 7.17 (dd, 3H, *J* = 8.19 Hz), 7.07−6.99 (m, 5H, *J* = 7.80 Hz), 6.90 (t, 1H, *J* = 5.07 Hz), 3.19−3.14 (m, 2H, *J* = 7.42 Hz), 2.67 (t, 2H, *J* = 7.41 Hz), 1.37 (s, 9H). HRMS (ESI, *m/z*): [M+H]^+^ calcd for C_29_H_31_N_2_O_2_^+^, 439.2386; found, 439.2379. [M+Na]^+^ calcd for C_29_H_30_N_2_O_2_Na^+^, 461.2205; found, 461.2195.

*Synthesis of P-TPEA-Boc*

*N*-Phenyl-4-biphenylamine (808.9 mg, 3.30 mmol), tris-(dibenzylideneacetone)dipalladium(0) (90 mg), compound **1** (1018 mg, 3.40 mmol), sodium *tert*-butoxide (220 mg), and *tri*-*tert*-butylphosphine tetrafluoroborate (26 mg) were dissolved into anhydrous toluene (5 mL) in a 50 mL two-necked flask. The reaction was heated to 120 °C for 18 h. After cooling to RT, the mixture was extracted with brine water and dichloromethane. The organic phase was collected and the solution was evaporated under reduced pressure. The crude product was purified by column chromatography (silica gel, EA/ PE = 1:10, v/v) to give a white solid (1010 mg, 66%).  ^1^H NMR (DMSO-*d*6, 400 MHz): *δ* 7.64 (d, 2H, *J* = 7.34 Hz), 7.59 (d, 2H, *J* = 8.44 Hz), 7.48−7.42 (m, 2H, *J* = 7.33 Hz), 7.31 (dd, 3H, *J* = 8.07 Hz), 7.17 (d, 2H, *J* = 8.08 Hz), 7.07−6.99 (m, 7H, *J* = 8.44 Hz), 6.90 (t, 1H, *J* = 5.50 Hz), 3.18−3.13 (m, 2H, *J* = 6.97 Hz), 2.66 (t, 2H, *J* = 7.34 Hz), 1.37 (s, 9H). HRMS (ESI, *m/z*): [M+Na]^+^ calcd for C_31_H_32_N_2_O_2_Na^+^, 487.2361; found, 487.2359.

*Synthesis of N-TPEAI*

N-TPEA-Boc (438.2 mg, 1.0 mmol) was dissolved in 100 ml ethyl acetate, and the reaction was cooled in ice bath for 1 h. Hydroiodic acid (2.0 mmol, ca. 55%) aqueous solution was dropped in the reaction and then the mixture was warmed to RT. After stirring for 2 h, the solution was evaporated under reduced pressure to obtain a white solid (327 mg, 70%). ^1^H NMR (DMSO-*d*6, 400 MHz): *δ* 7.97 (s, 3H), 7.85−7.82 (m, 2H, *J* = 4.63 Hz), 7.68 (d, 1H, *J* = 7.98 Hz), 7.44−7.38 (m, 3H, *J* = 9.24 Hz), 7.33 (dd, 2H, *J* = 7.98 Hz), 7.21 (dd, 3H, *J* = 8.40 Hz), 7.09−7.02 (m, 5H, *J* = 7.99 Hz), 3.06 (t, 2H, *J* = 7.14 Hz), 2.85 (t, 2H, *J* = 8.41 Hz).

*Synthesis of P-TPEAI*

P-TPEA-Boc (464.2 mg, 1.0 mmol) was dissolved in 100 ml ethyl acetate, and the reaction was cooled in ice bath. Hydroiodic acid (2.0 mmol, ca. 55%) aqueous solution was dropped in the reaction and then the mixture was warmed to RT. After stirring for 2 h, the solution was evaporated under reduced pressure to obtain a pale yellow solid (354 mg, 72%). ^1^H NMR (DMSO-*d*6, 400 MHz): *δ* 8.02 (s, 3H), 7.66−7.58 (m, 4H, *J* = 5.85 Hz), 7.44 (dd, 2H, *J* = 7.31 Hz), 7.33 (dd, 3H, *J* = 7.31 Hz), 7.24 (d, 2H, *J* = 8.40 Hz), 7.09−7.03 (m, 7H, *J* = 8.04 Hz), 3.05 (t, 2H, *J* = 8.05 Hz), 2.86 (t, 2H, *J* = 8.41 Hz).

3. Device Fabrication

All the solar cell fabrication processes were conducted in ambient conditions (RH: 20%–30%, room temperature). The FTO glass substrates were cleaned with detergent, deionized water, acetone, and ethanol, respectively. The SnO_2_ electron transport layer was deposited onto the FTO substrates by a chemical bath deposition (CBD) method [3]. CBD solution was prepared by dissolving urea (625 mg), HCl (37 wt%, 625 μL), thioglycolic acid (12.5 μL), and SnCl_2_·2H_2_O (137.5 mg) in 50 mL deionized water. The cleaned FTO substrates were soaked in CBD solution, and heated at 88 °C for 4 h. Then, the SnO_2_-deposited FTO subtrates were ultrasonically cleaned with deionized water and IPA, followed by annealing at 170 °C for 1 h. Subsequently, the subtrates were spin-coated with KCl aqueous solution (10 mmol/mL) at 3000 rpm for 30 s and annealed at 100 °C for 10 min.

The FAPbI_3_ films were fabricated according to a previous report [3]. The perovskite precursor solution was composed of 1.53 M PbI_2_, 1.4 M FAI, 0.5 M MACl, and 0.0122 M MAPbBr_3_ in 1 mL DMF and DMSO mixed solution (v/v, 8:1). After the SnO_2_/FTO substrates were treated with oxygen plasma, the perovskite films were formed on SnO_2_ by spin-coating 40 μL of perovskite precursor solution at 2000 rpm for 10 s and 6000 rpm for 30 s. During the second spin-coating step, 200 μL of chlorobenzene was dripped onto the substrates after 10 s. The obtained perovskite films were annealed at 100 °C for 50 min, then cooling down to ca. 60 °C for 30 min. Perovskite films should be cooled down to room temperature before other operations. For 2D/3D perovskites, N-TPEAI or P-TPEAI (2−4 mg/ mL) in chloroform (CF) were spin-coated on the perovskite films at 5000 rpm for 30 s, and then the films were annealed at 120 °C for 7 min. Subsequently, PTAA (30 mg/ mL) solution doped with 11% TPFB (mass ratio) in chlorobenzene was spin-coated on the perovskite films at 4000 rpm for 30 s, followed by annealing at 80 °C for 5 min [4]. Finally, 100-nm-thick gold counter electrode was deposited by thermal evaporation.

4. Characterizations

^1^H NMR spectra were measured on Bruker spectrometers at 400 MHz (Fällanden, Switzerland). Chemical shifts were calibrated with tetramethylsilane (TMS) as the internal standard. UV-vis absorption spectra were recorded by an Agilent 8453 spectrophotometer. Steady state and time-resolved photoluminescence spectra were collected by FLS1000 (Edinburgh, UK) with *λ*_exaction_ = 450 nm and *λ*_emission_ = 800 nm. The lifetimes were determined by fitting the kinetics with bi-exponential function: *Y* = *A*_1_exp(–*t*/*τ*_1_) + *A*_2_exp(–*t*/*τ*_2_) + *y*_0_. The 2D-GIWAXS was performed at BL17B1 and BL02U2 beamline of SSRF using the X-ray wavelength of 1.24 Å. Two-dimensional patterns were acquired by a PLATUS 2M detector mounted vertically at a distance ~306 mm from the sample with a grazing incidence angle of 0.3° and an exposure time of 10 s.

X-ray photoelectron spectroscopy (XPS) were obtained by using ESCALAB250Xi (Thermo Scientific, UK) and ultraviolet photoelectron spectroscopy (UPS) was measured using a VGScientaR4000 analyzer and a HeI discharge lamp (21.22 eV). The work functions (*W*_F_) and valence band maximum (VBM) were determined from the cutoff and Fermi edge of UPS spectra by using the equations: *W*_F_ = 21.22 eV − *E*_cutoff_ and VBM = 21.22 eV − (*E*_cutoff_ − *E*_edge_). The top-view and cross-sectional SEM images were characterized with a scanning electron microscope (SEM, JEOL JSM-7900F). Atomic force microscopy (AFM), conductive atomic force microscopy (C-AFM), and Kelvin probe force microscopy (KPFM) were measured by an atomic force microscopy (JPK Nanowizard 4XP, Bruker).

The cyclic voltammetry (CV) measurements were performed with a CHI 660E electrochemistry workstation. The redox potentials of N-TPEA-Boc and P-TPEA-Boc were measured in acetonitrile with tetrabutylammonium hexafluorophosphate (TBAPF_6_, 0.1 M) as the supporting electrolyte. A glassy carbon electrode with a surface area of 0.785 cm^2^ was used as the working electrode. The counter electrode was a platinum wire and an Ag/AgCl electrode was used as the reference electrode. The redox potentials were calibrated with Fc/Fc^+^ as an external standard. The HOMOs of N-TPEA-Boc and P-TPEA-Boc were measured according to a previously reported method [4].

The *J*–*V* characteristics of the devices were recorded by using a Keithley 2400 under 100 mW cm^−2^ (AM 1.5G) (Newport USA, 94023A). The light intensity was calibrated each week by a standard Si reference cell (SER. No: 506/0358) certified by National Renewable Energy Laboratory (NREL). *J*−*V* curves were measured in a glove box filled with nitrogen. A mask with an aperture (0.075 cm^2^) smaller than the active area of the square solar cell (0.20 cm^2^) was applied on top of the devices. The *J*–*V* curves were obtained by the reverse scan (1.35 V to − 0.2 V). The step voltage and delay time were fixed at 50 mV and 200 ms, respectively. For the measurements of the hysteresis, the *J*–*V* curves were recorded through forward scan (–0.2 V – 1.35 V) or reverse scan (1.35 V – –0.2 V) directions. The external quantum efficiency (EQE) measurements were obtained by TLS-300XR (Newport) without background illumination. After treated by background, the integral current density (*J*_int_) are calculated with the equation: $J_{\mathrm{int}}= q\int_{{}_{\min}}^{{}_{\max}} IPCE (){}_{\mathrm{photon}}()d$, in which *q* is electronic charge, *λ* is wavelength, *IPCE*(*λ*) is obatined from EQE data, *Φ*_photon_(*λ*) is photon flux density spectrum of a standard light source (obtained from NREL, AM 1.5G, 100 mW cm^−2^). The transient photocurrent (TPC) decay, the transient photovoltage (TPV), Mott-Schottky plots, and the linear relationship between *V*_oc_ and light intensity was characterized by PAIOS 3.2.

The SCLC characterization was performed with hole-only devices (FTO/PEDOT:PSS/perovskite/PTAA/Ag), and the voltage was scanned from 0 to 5 V with step size of 20 mV under a dark condition. The hole mobility was calculated by fitting the curve using Mott-Gurney law in Child’s regime with the equation *J*(*V*) = 9*εε*_0_*μV*^2^/8*d*^3^, where *ε* is the dielectric constant of the material (normally taken to approach 14 for perovskites), *μ* is the hole mobility, *V* is the applied bias, and *d* is the film thickness (640 nm). The defect density was calculated using equation *N*_t_ = 2*ε*_0_*ε*_r_*V*_TFL_/*qd*^3^, where *V*_TFL_ is the onset voltage of the trap filled limit region and *q* is the elemental charge.

Fill factor (FF) loss in the best-performing devices were determined by two main factors, non-radiative loss and charge transport loss [5, 6]. The maximum FF (FF_max_) can be empirically calculated according to the following equation:

$$FFmax= \frac{v_{\mathrm{oc}}-\ln(v_{\mathrm{oc}}+0.72)}{v_{\mathrm{oc}}+1}$$

where $v_{oc}= \frac{V_{\mathrm{oc}}}{n_{\mathrm{id}}K_{B}T/q}$, *n*_id_ is ideality factor, *K*_B_ is Boltzmann constant, *T* is temperature, *q* is elementary charge. The ideality factors were extracted from the *V*_oc_ as a function of light intensity on a logarithmic scale (Fig. S30).

The MPP tracking stability tests of PSCs without encapsulations were conducted under continuous 1-sun illumination (100 mW cm^−2^, AM 1.5 G, MT-PV-16, Tianjin Meto), following the ISOS-L-2 testing protocol (Light source: LED lamp; temperature: 65°C; atmosphere: nitrogen; load: MPP). The device area for the stability test is 0.04 cm^2^. The step voltage was 20 mV. The *J*–*V* curves were automatically recorded with a reverse scan each hour. The MPP stability tests were implemented without active cooling and the devices were performed at 65°C.

5. Calculational Method

All calculations regarding the structures in this study were performed using density functional theory (DFT) with the generalized gradient approximation (GGA) of the Perdew-Burke-Ernzerhof (PBE) exchange-correlation functional and the projector augmented-wave (PAW) pseudopotential method, as implemented in the VASP software package [7−9]. The plane-wave basis set was truncated at an energy cutoff of 500 eV. Electronic self-consistency was reached when the total energy change fell below 1×10^-5^ eV, and ionic relaxations proceeded until all forces were less than 0.05 eV Å⁻¹ after simultaneous optimization of both atomic positions and cell parameters. To accurately capture the effects of weak interactions, both spin polarization and spin–orbit coupling (SOC) were included in the calculations. Additionally, the Grimme DFT-D3 [10] dispersion correction with a zero-damping function was applied to account for van der Waals interactions. Formation enthalpies at 0 K were computed as [11]:

$$\Delta H_{f}^{0\text{K}}=E\left[ A_{2}PbI_{4} \right]-\left( 2E\left[ \text{AI} \right]+E\left[ \text{Pb}\text{I}_{\text{2}} \right] \right)$$

where $E\left[ A_{2}PbI_{4} \right]$, $E\left[ \text{AI} \right]$ and $E\left[ \text{Pb}\text{I}_{\text{2}} \right]$ are the total energies of the perovskite, the organic halide precursor, and lead diiodide, respectively-each obtained from fully relaxed structures under the above convergence criteria.

All quantum chemical calculations were performed using the Gaussian 09 software package [12]. The geometry optimizations and subsequent electronic structure analyses were carried out at the B3LYP/6-311G level of theory [13, 14]. To account for dispersion interactions, the Grimme’s DFT-D3 empirical dispersion correction with Becke–Johnson damping was applied throughout the calculations [15]. Binding energies were computed as the difference in total energies between the optimized complex and its constituent monomers, with basis set superposition error (BSSE) correction applied using the counterpoise method where relevant [16]. The binding energy (*E*_b_), representing the energy required to dissociate molecule A from molecule B, was computed as follows,

*E*_b_ = *E*_(A/B)_ − *E*_A_ − *E*_B_

Hole transfer integral (HTI) was calculated as half of the energy gap between the HOMO and HOMO−1 for the combination states [17−19].

HTI = (*I*_X_ − *I*_N_)/2 = (*ε*_N_ − *ε*_X_)/2

where *I*_i_ and *ε*_i_ (i = X and N) are the ionization potential and the eigenvalue of the canonical MO *ϕ*_i_ (i = X and N), respectively. When *ϕ*_X_ is HOMO−1, HTI in such cases is given by

HTI = (*E*_HOMO_ − *E*_HOMO−1_)/2

6. Supplementary Figures

**Fig. S1** ^1^H NMR spectrum of N-TPEA-Boc (DMSO-*d6*, 400 MHz).

**Fig. S2** HRMS spectrum of N-TPEA-Boc.

**Fig. S3** ^1^H NMR spectrum of P-TPEA-Boc (DMSO-*d*6, 400 MHz).

**Fig. S4** HRMS spectrum of P-TPEA-Boc.

**Fig. S5** ^1^H NMR spectrum of N-TPEAI (DMSO-*d*6, 400 MHz).

**Fig. S6** ^1^H NMR spectrum of P-TPEAI (DMSO-*d*6, 400 MHz).

**
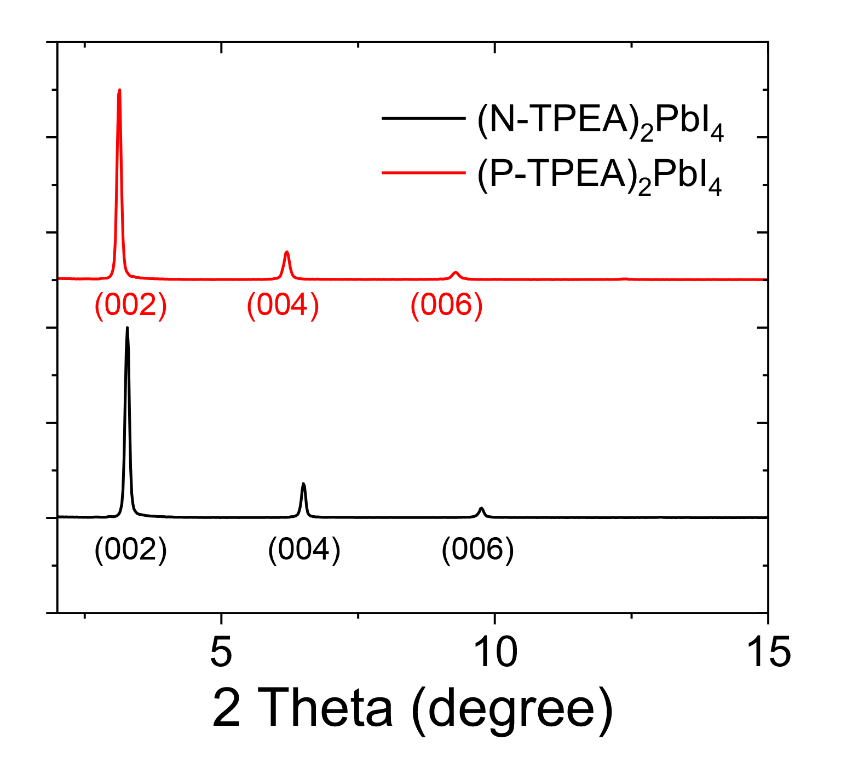
**

**Fig. S7** XRD patterns of (N-TPEA)_2_PbI_4_ and (P-TPEA)_2_PbI_4_ 2D perovskite films.

**
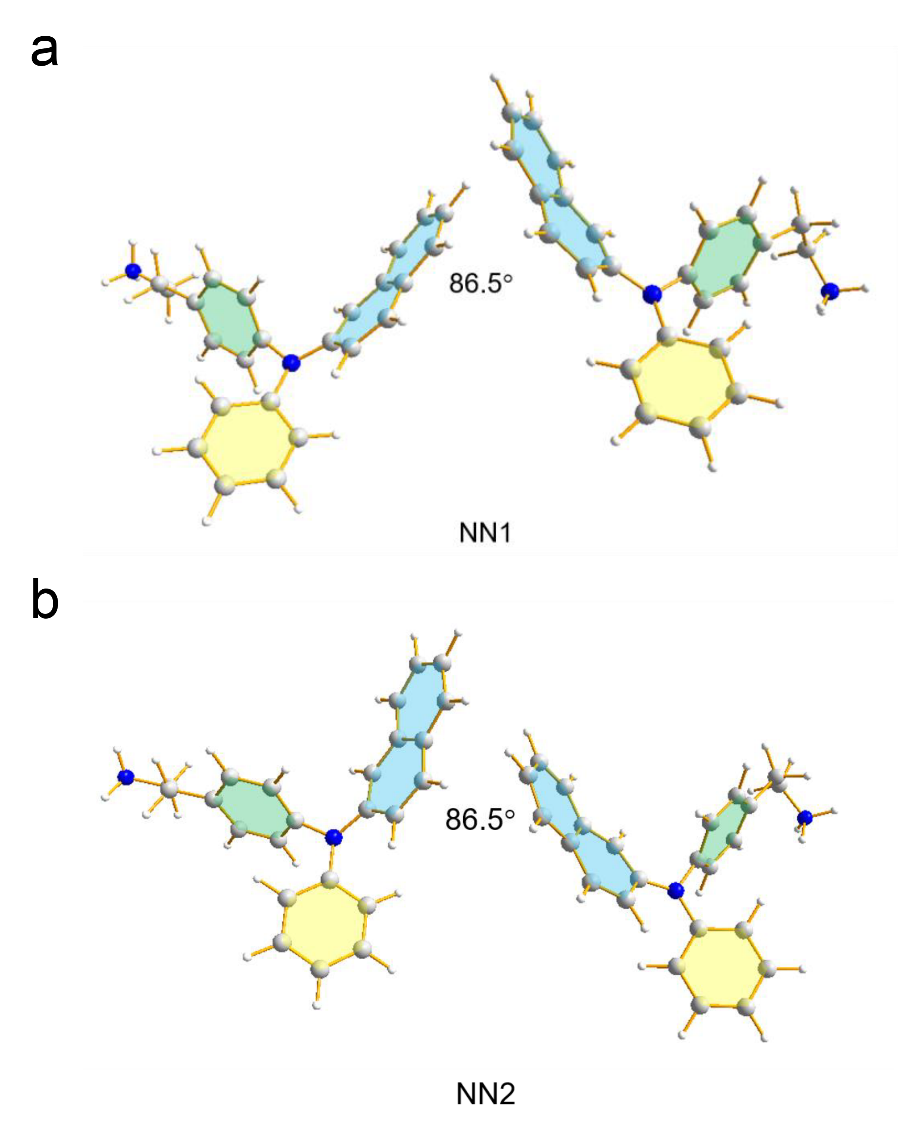
**

**Fig. S8** Schematic illustration of the geometrical structures of N-TPEA^+^ cation pairs in (N-TPEA)_2_PbI_4_ 2D perovskites from DFT calculations.


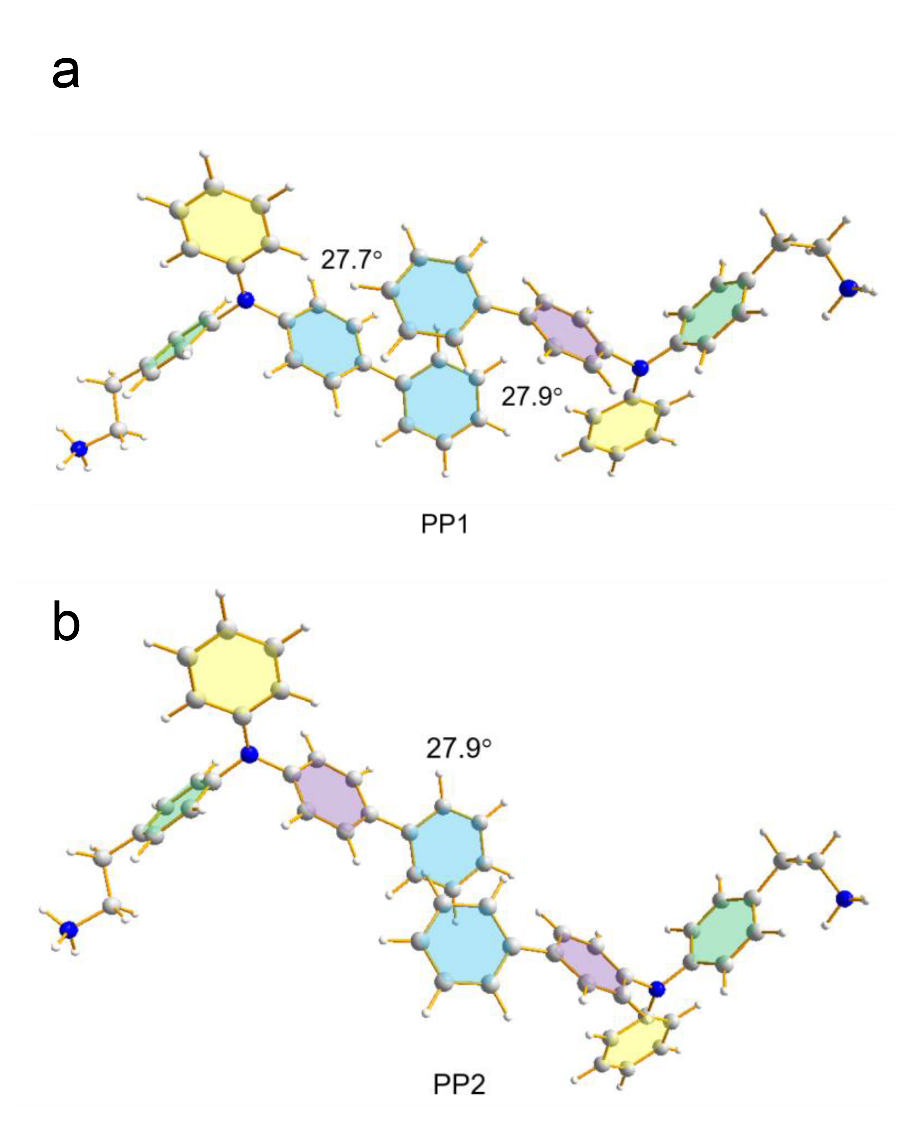


**Fig. S9** Schematic illustration of the geometrical structures of P-TPEA^+^ cation pairs in (P-TPEA)_2_PbI_4_ 2D perovskites from DFT calculations.


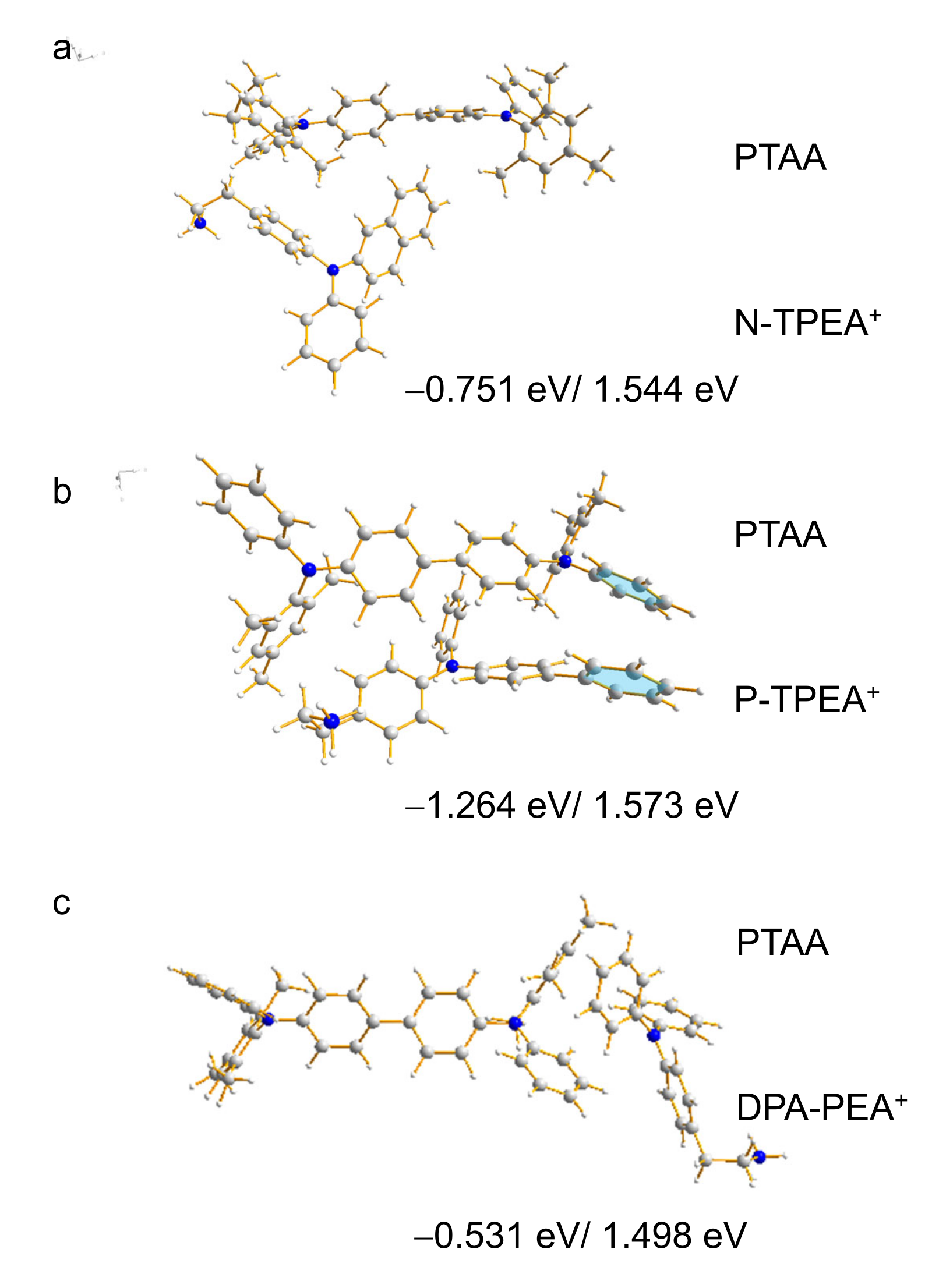


**Fig. S10** Calculated binding energy and hole transfer integral of **a** N-TPEA^+^/PTAA, **b** P-TPEA^+^/PTAA, and **c** DPA-PEA^+^/PTAA.

**
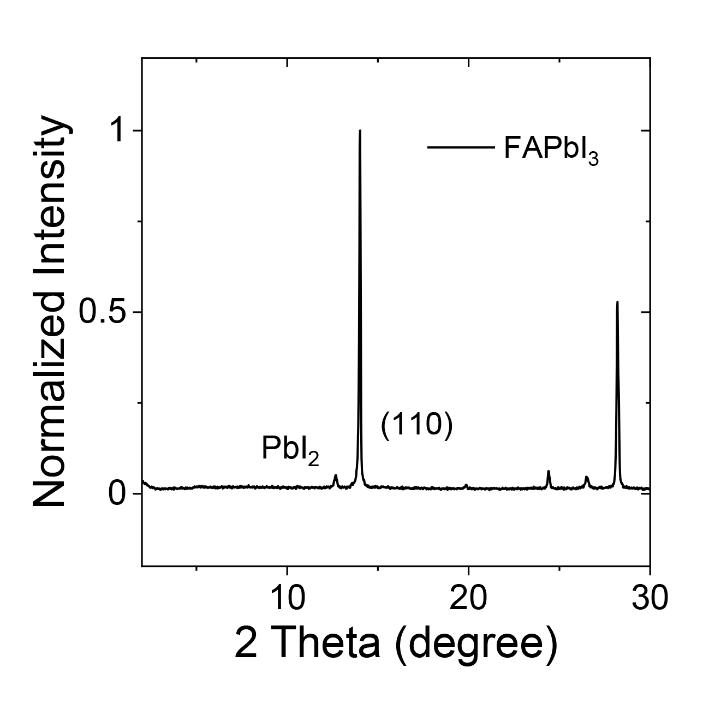
**

**Fig. S11** XRD pattern of FAPbI_3_ film.


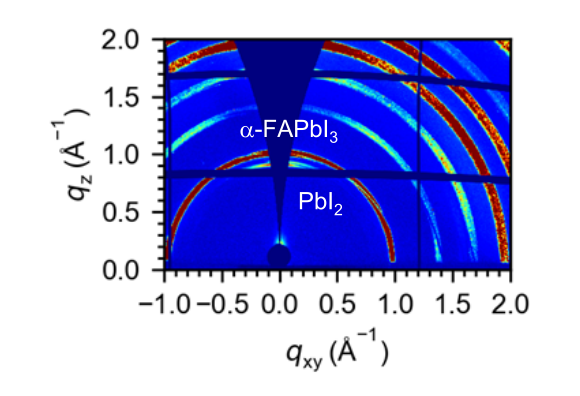


**Fig. S12** 2D GIWAXS pattern of FAPbI_3_ film.


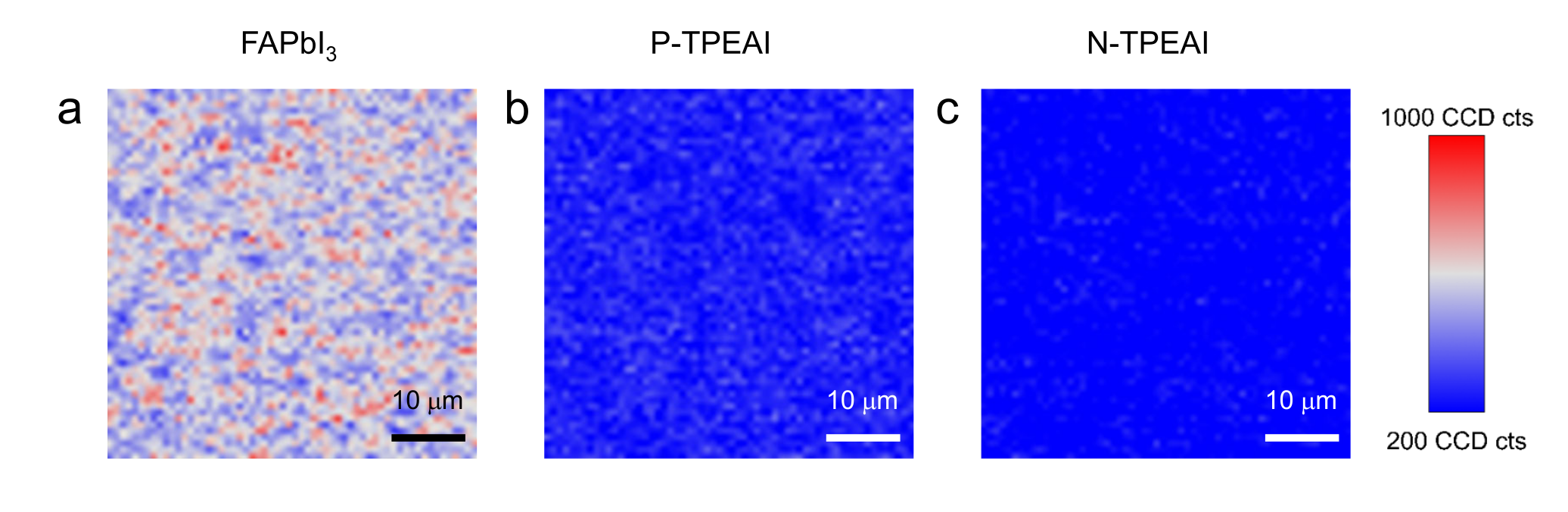


**Fig. S13** PL mapping of FAPbI_3_, P-TPEAI-treated, and N-TPEAI-treated perovskite films.


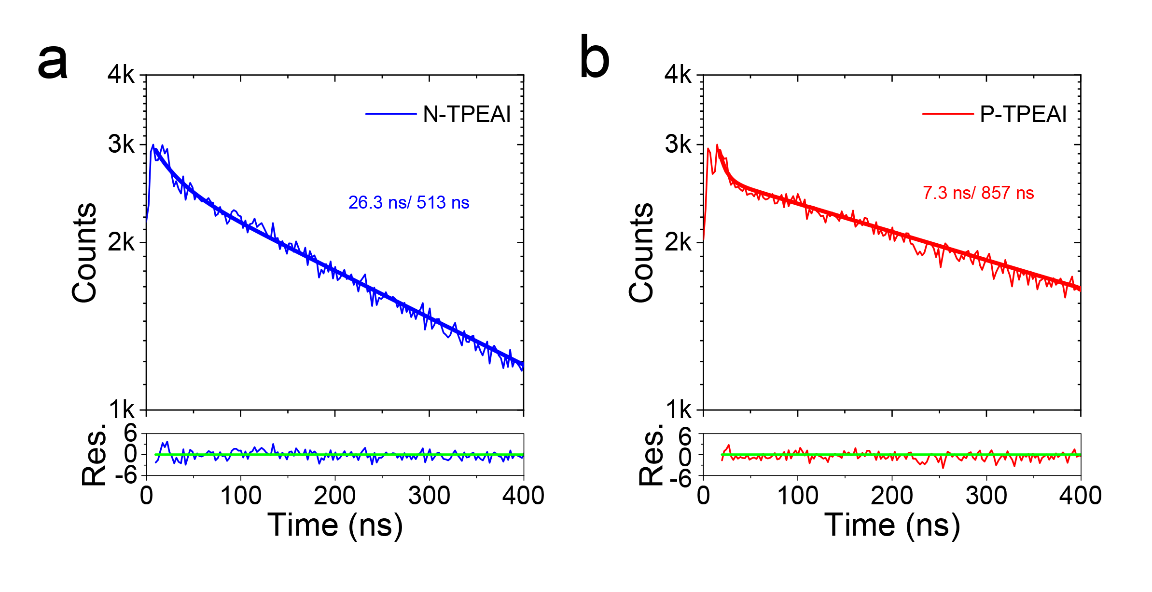


**Fig. S14** The partial enlarged details of TRPL kinetics at the initial stage of **a** N-TPEAI and **b** P-TPEAI treated perovskites with residual errors.


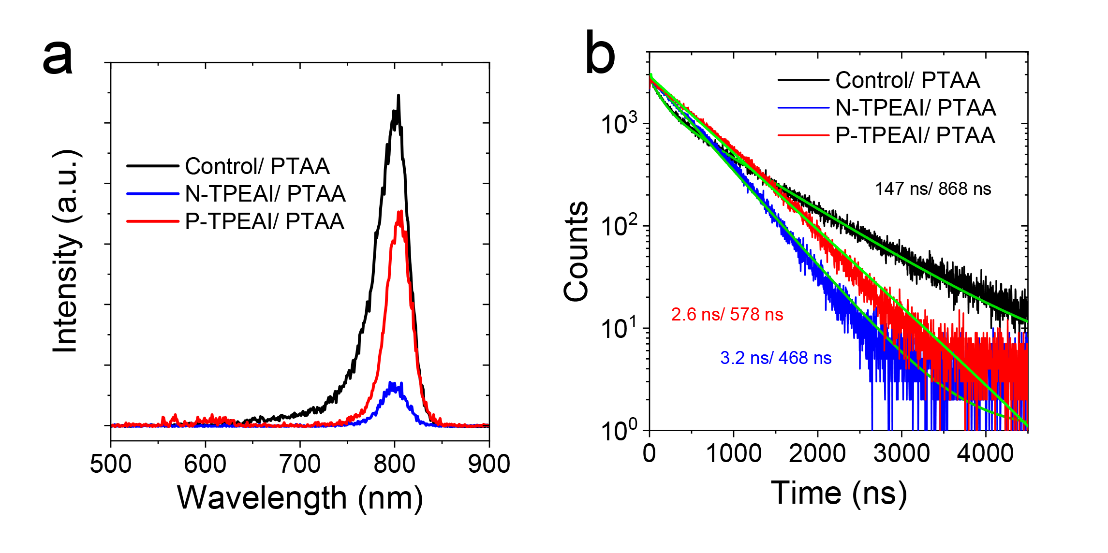


**Fig. S15 a** Steady-state PL and **b** TRPL kinetics of pristine and treated perovskite films with PTAA as HTLs.

**
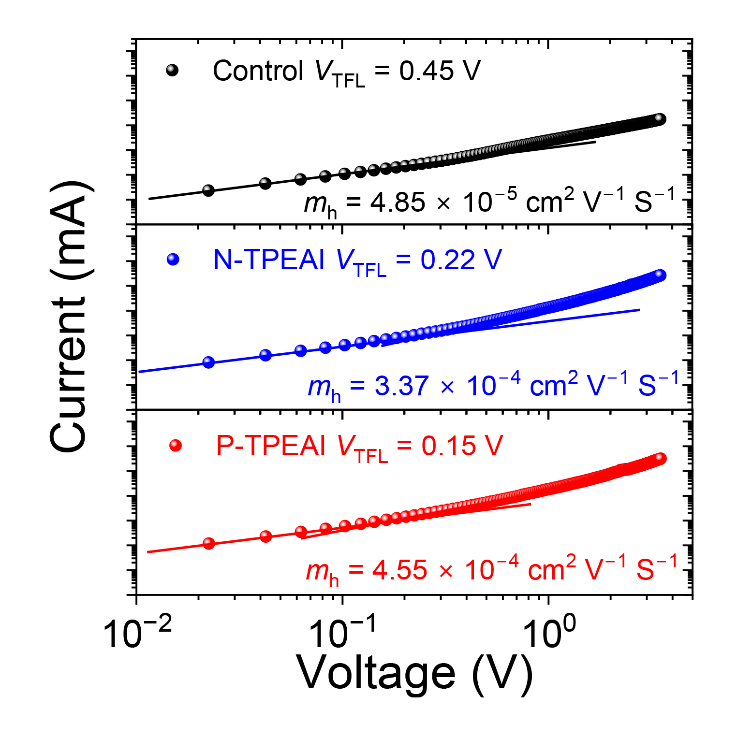
**

**Fig. S16** *I*−*V* curves of the hole-only devices measured under dark conditions.

**
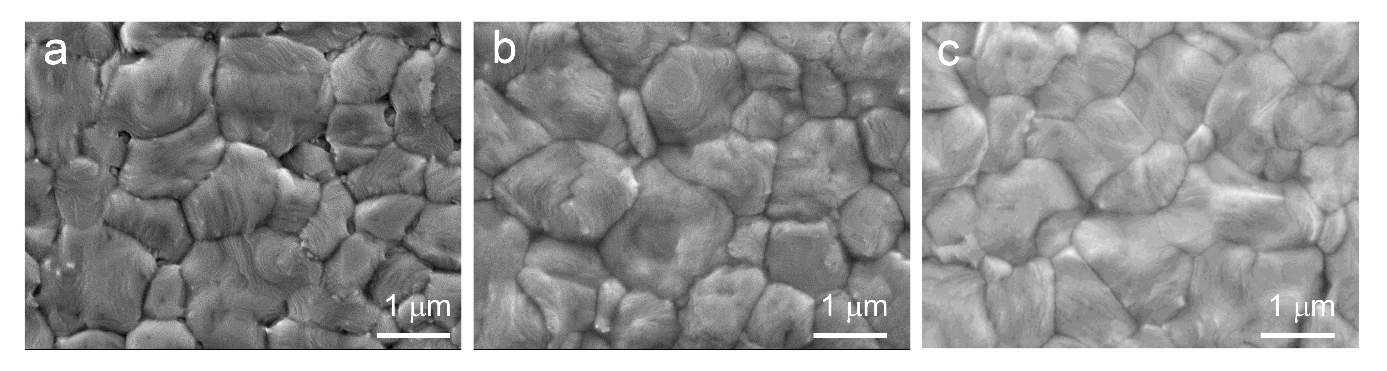
**

**Fig. S17** Top-view SEM images of **a** control, **b** N-TPEAI-treated, and **c** P-TPEAI-treated perovskite films.

**
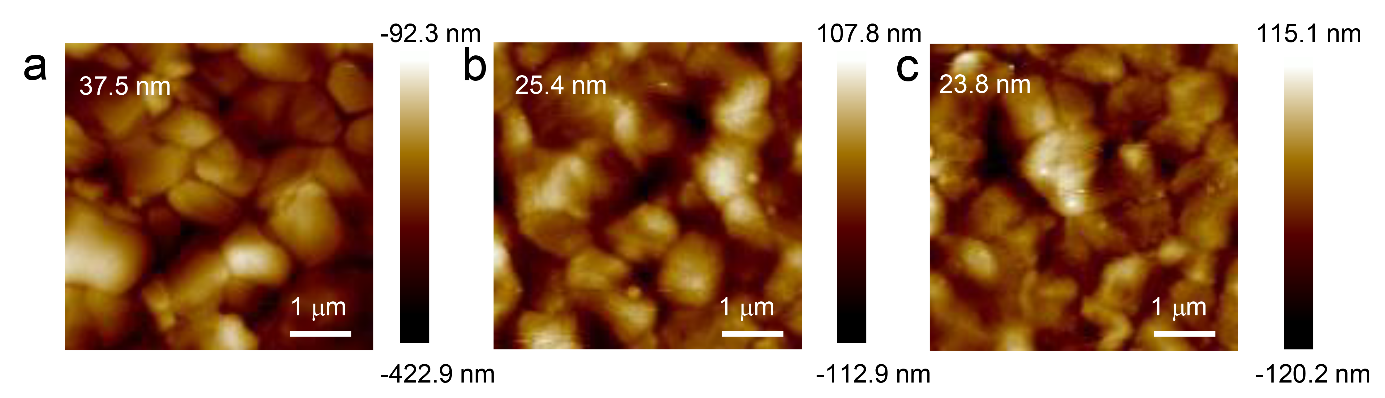
**

**Fig. S18** AFM images of **a** control, **b** N-TPEAI-treated, and **c** P-TPEAI-treated perovskite films.


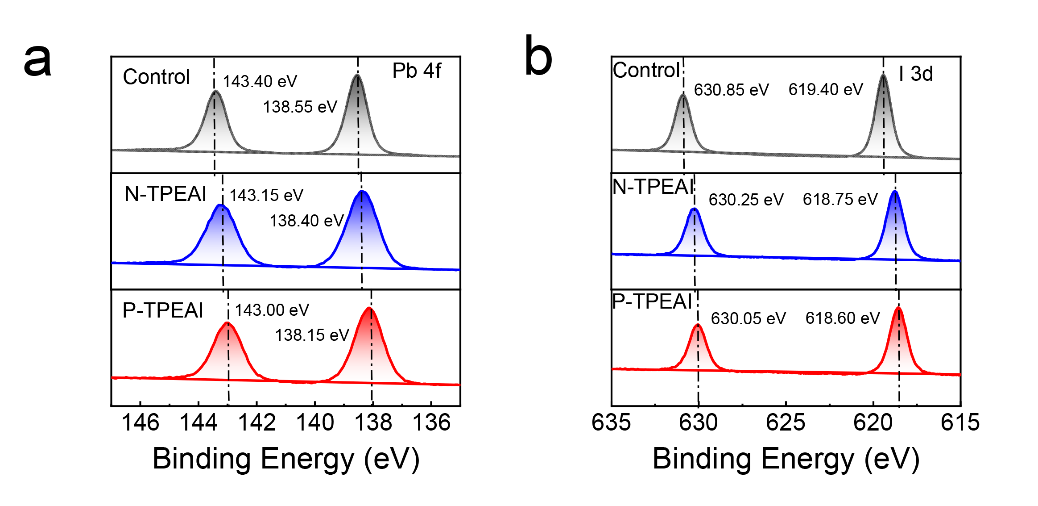


**Fig. S19** XPS spectra of perovskite films with and without treatment, and the details of peaks are presented.

**
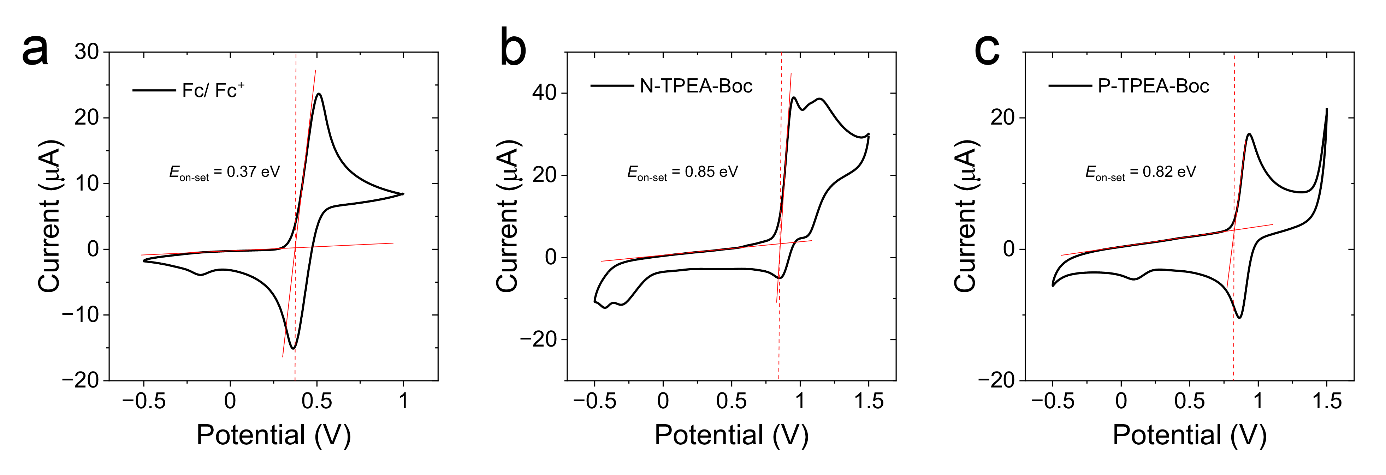
**

**Fig. S20** Cyclic voltammograms of **a** Fc/ Fc^+^ (Fc = Ferrocene), **b** N-TPEA-Boc, and **c** P-TPEA-Boc in acetonitrile, potential versus Ag/AgCl.

**
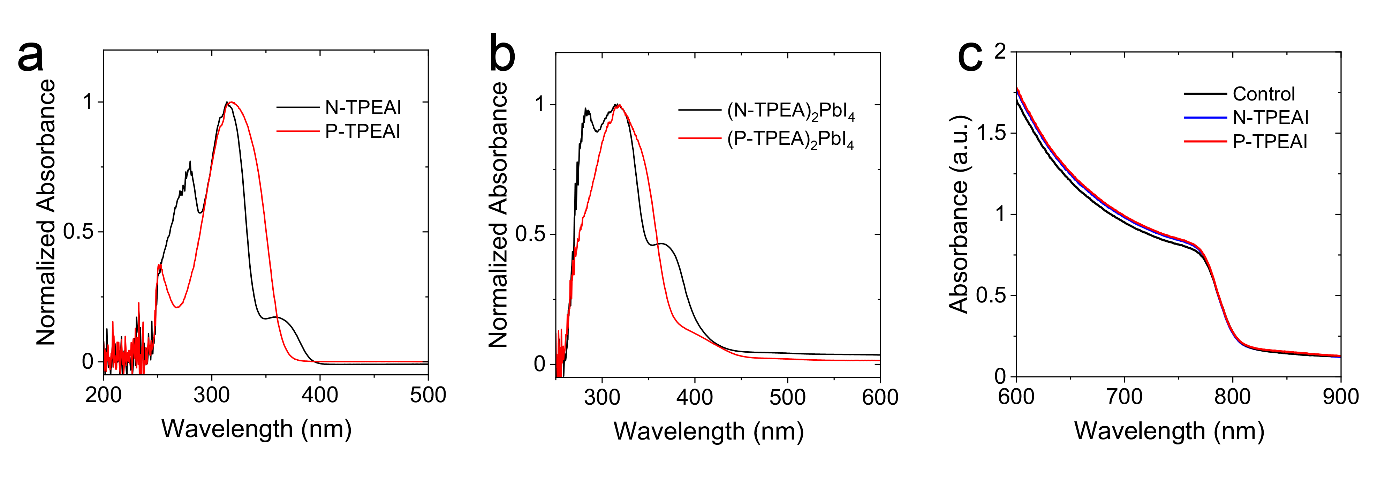
**

**Fig. S21** Normalized UV-vis absorption spectra of **a** N-TPEAI and P-TPEAI in chloroform; **b** pure (N-TPEA)_2_PbI_4_ and (P-TPEA)_2_PbI_4_ 2D perovskites on the glass substrates. **c** UV-vis absorption spectra of pristine, N-TPEAI-treated, and P-TPEAI-treated perovskite films.

**
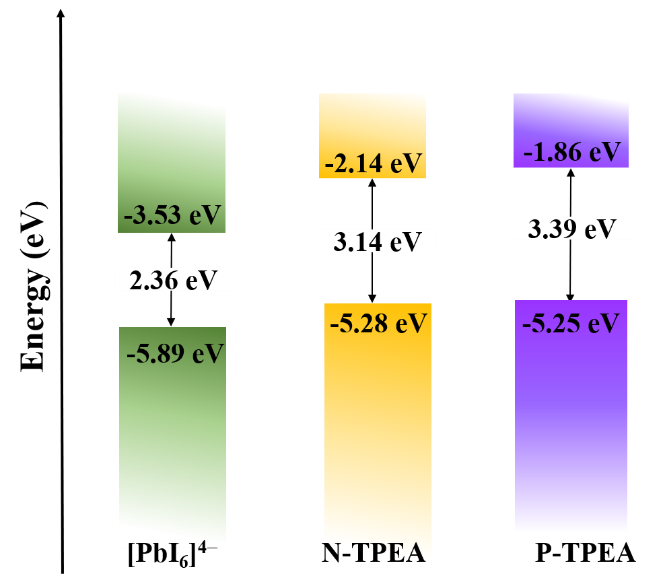
**

**Fig. S22** The schematic of the energy levels of N-TPEA and P-TPEA ligands with inorganic layer [PbI_6_]^4−^.


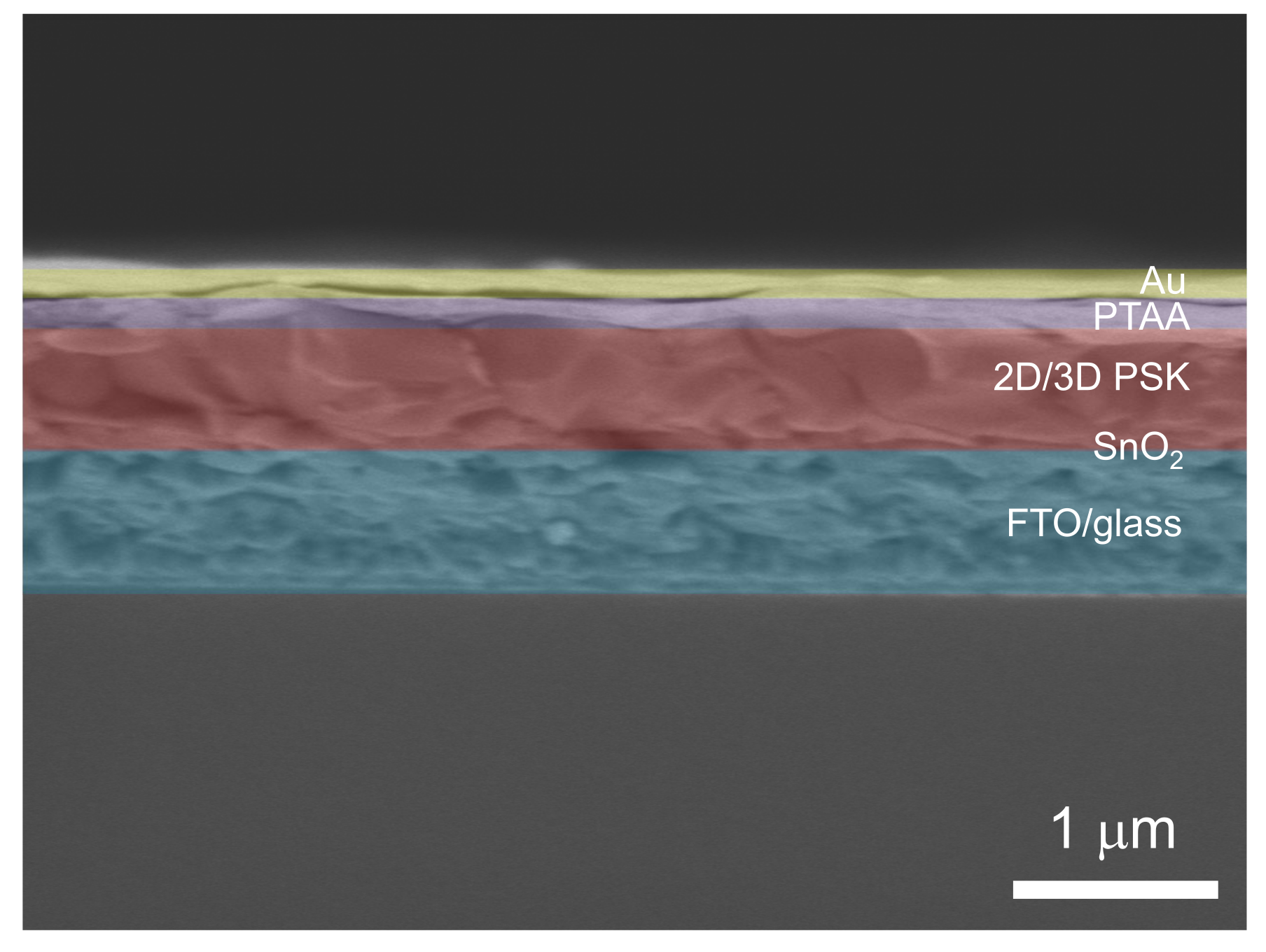


**Fig. S2****3** Cross-sectional SEM image of P-TPEAI-treated PSCs. The different functional layers are delineated with colored bands for clarity.

**
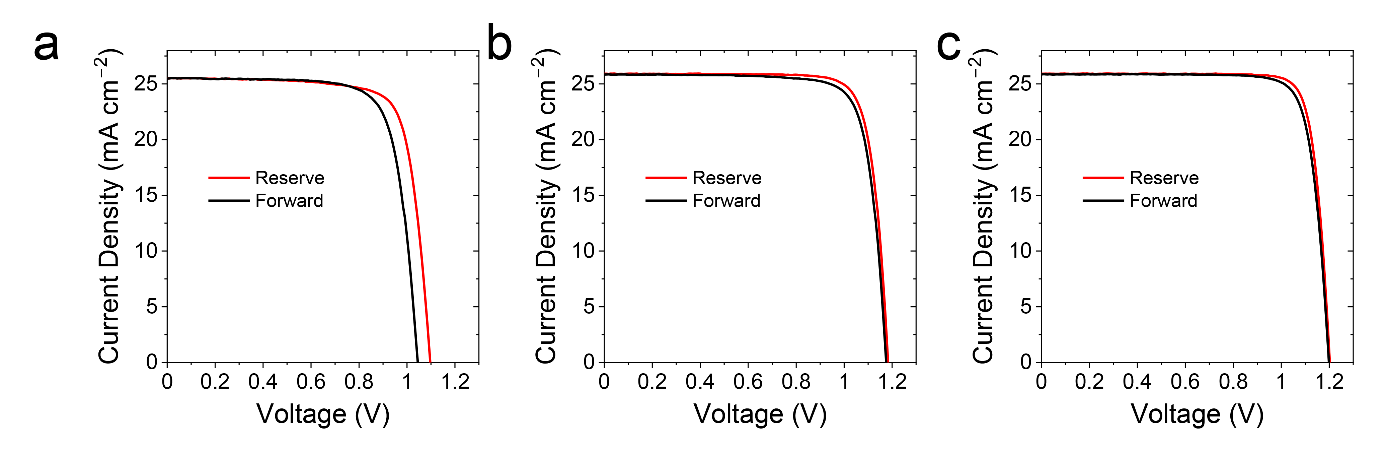
**

**Fig. S24** *J–V* curves of **a** control, **b** N-TPEAI-treated, and **c** P-TPEAI-treated PSCs measured at different scan directions under 100 mW cm^‒2^ (AM 1.5G).


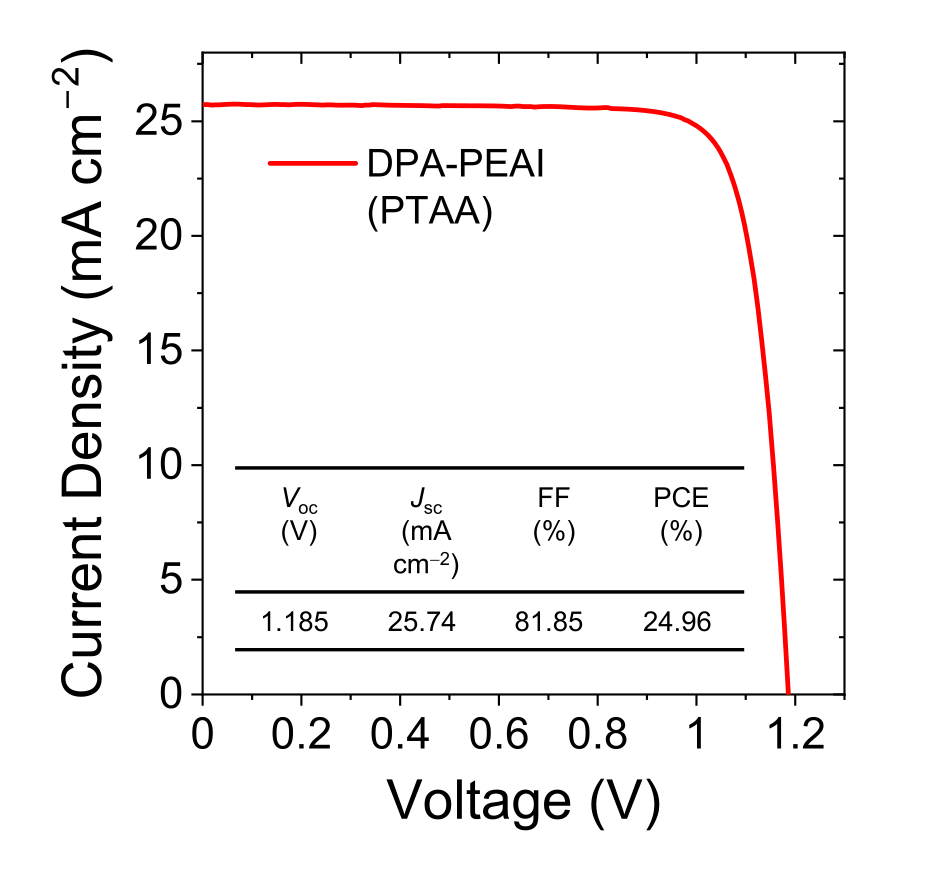


**Fig. S25** *J–V* curves of the best-performing DPA-PEAI-treated PSCs with PTAA as HTL measured under 100 mW cm^‒2^ (AM 1.5G).


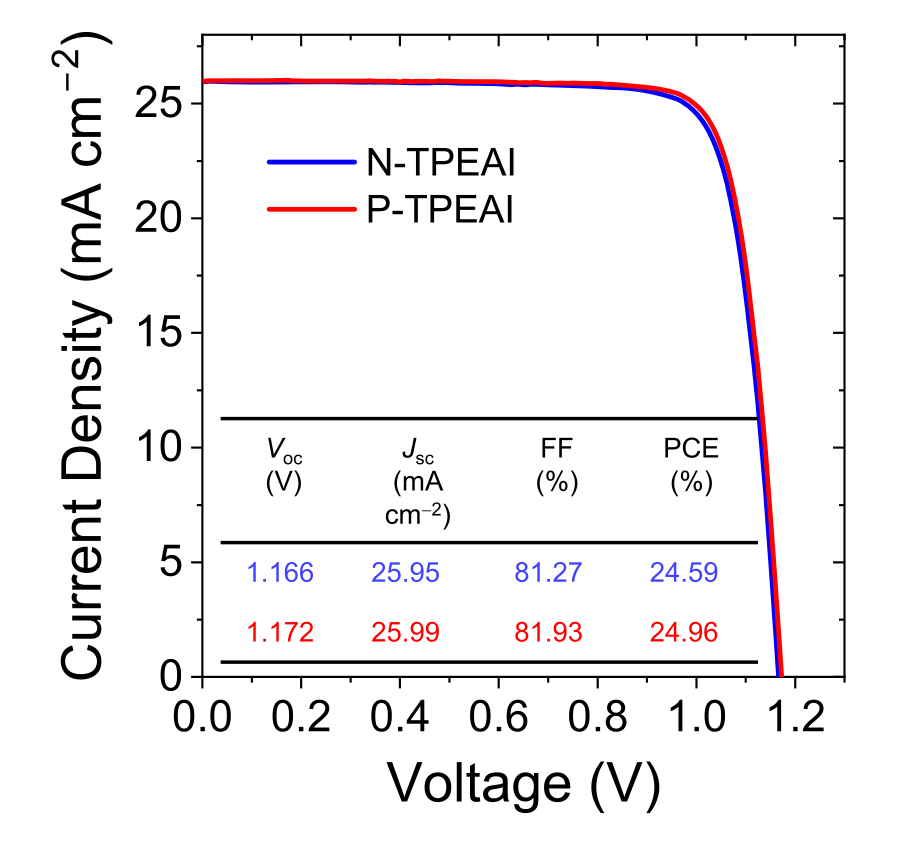


**Fig. S26** *J*−*V* curves characteristics of the N-TPEAI or P-TPEAI treated Spiro-OMeTAD based devices measured under 100 mW cm^‒2^ (AM 1.5G).

**
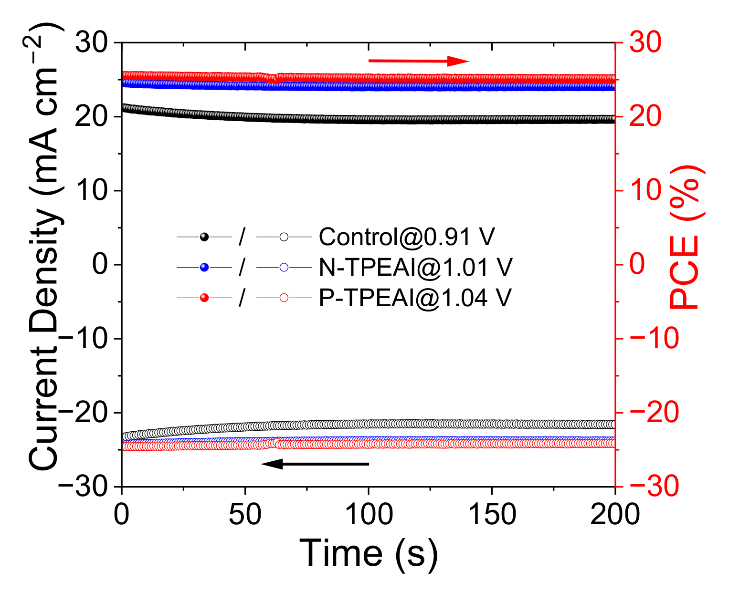
**

**Fig. S27** Steady-state tests of PCE and *J*_int_ at maximum power point voltage of PSCs. The stabilized PCEs of control, N-TPEAI-treated and P-TPEAI-treated devices are 21.19%, 24.59%, and 25.54%, respectively, with the correspounding current density of 23.29, 24.35, and 24.55 mA cm^−2^.


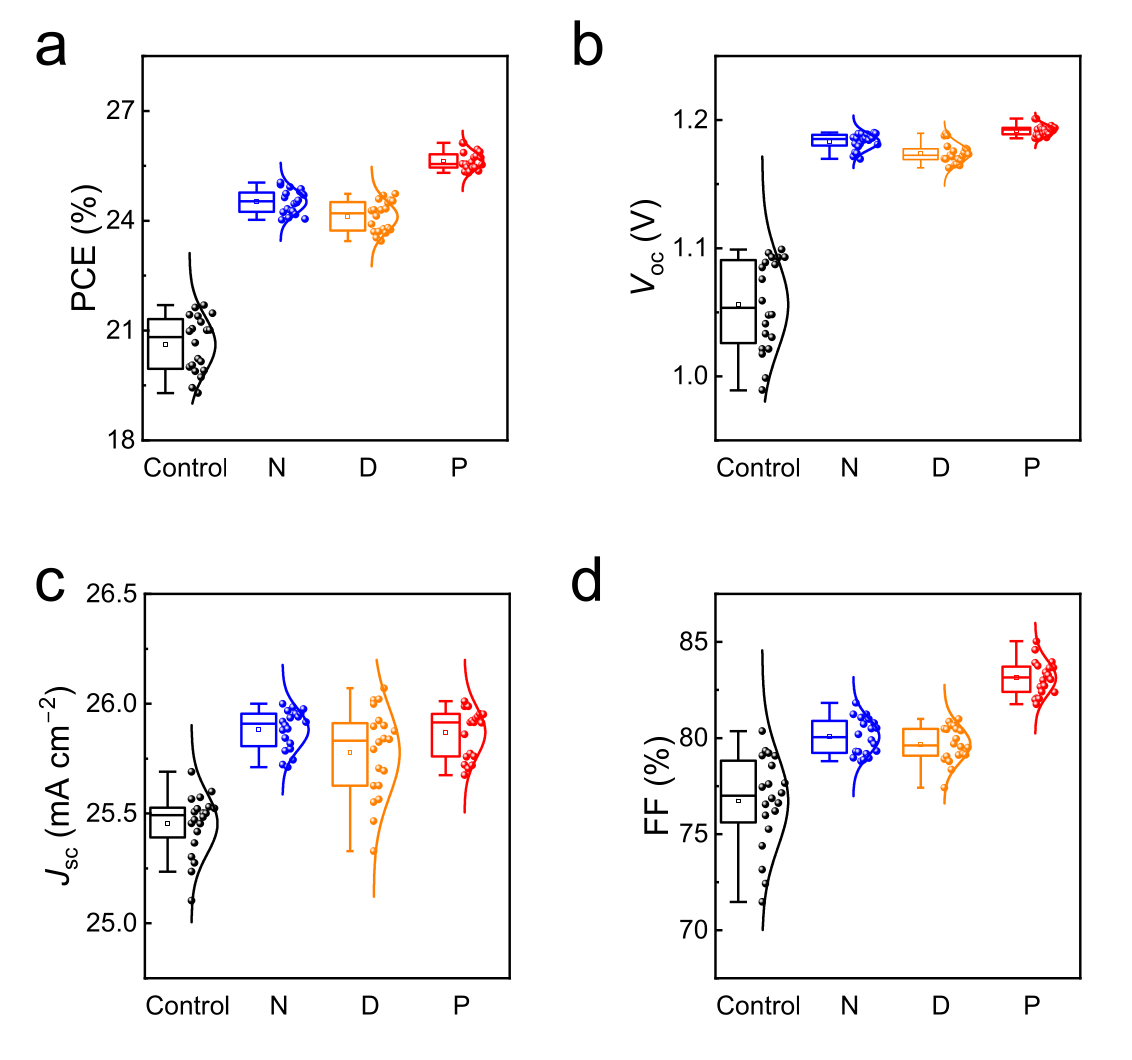


**Fig. S28** Statistical boxplots of PCE, *V*_oc_, *J*_sc_, and FF values of 20 PSCs (made in one run), in which N, D, P represent N-TPEAI, DPA-PEAI and P-TPEAI treated devices. The average PCEs of control, N-TPEAI-treated, DPA-PEAI-treated and P-TPEAI-treated devices are 20.61 ± 0.75%, 24.52 ± 0.32%, 24.11 ± 0.42%and 25.64 ± 0.25%, respectively, with the average *V*_oc_ of 1.056 ± 0.035 V, 1.183 ± 0.006 V, 1.174 ± 0.008 V and 1.192 ± 0.004 V, the average *J*_sc_ of 25.45 ± 0.14 mA cm^−2^, 25.88 ± 0.09 mA cm^−2^, 25.78 ± 0.20 and 25.87 ± 0.11 mA cm^−2^, the average FF of 76.72 ± 2.35%, 80.07 ± 0.93%, 79.68 ± 0.95% and 83.13 ± 0.89%.

**
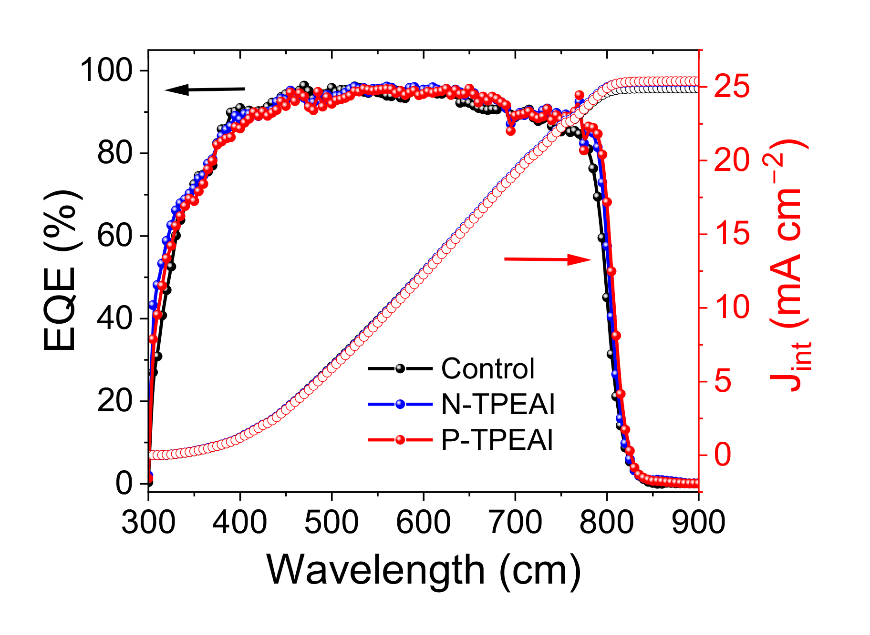
**

**Fig. S29** EQE spectra of PSCs and the corresponding integrated *J*_sc_. The integrated *J*_sc_ of control, N-TPEAI-treated and P-TPEAI-treated devices are 24.90 mA cm^−2^, 25.34 mA cm^−2^, and 25.38 mA cm^−2^, respectively (within 5% deviation).

**
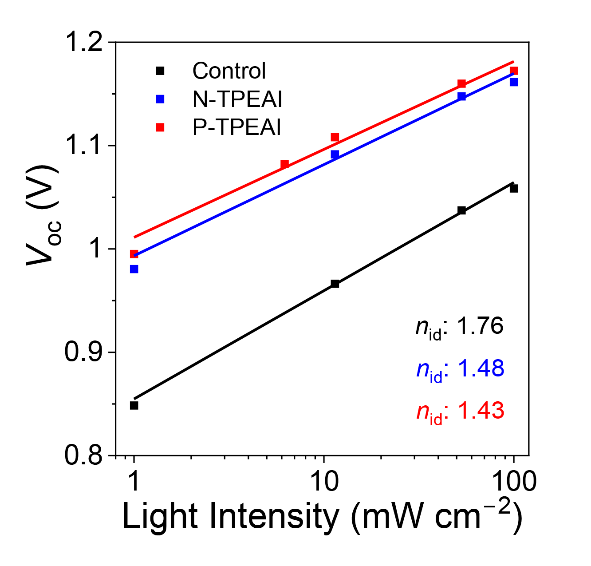
**

**Fig. S30** Light intensity dependence of *V*_oc_ for PSCs with and without treatment. The ideality factor *n*_id_ is derived from the slope of the linear fit of the semilogarithmic plot.


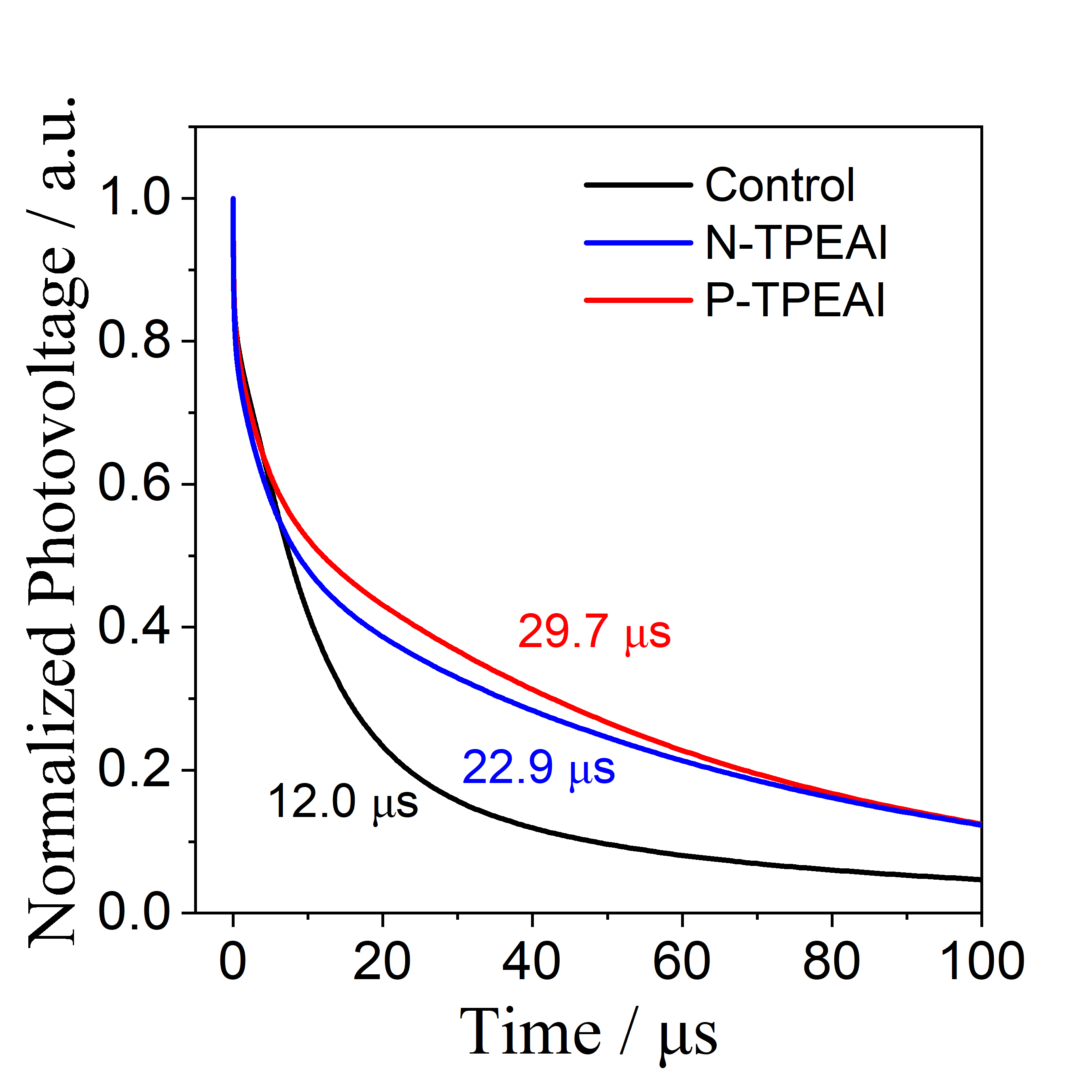


**Fig. S31** Transient photovoltage decay of PSCs with and without treatment.


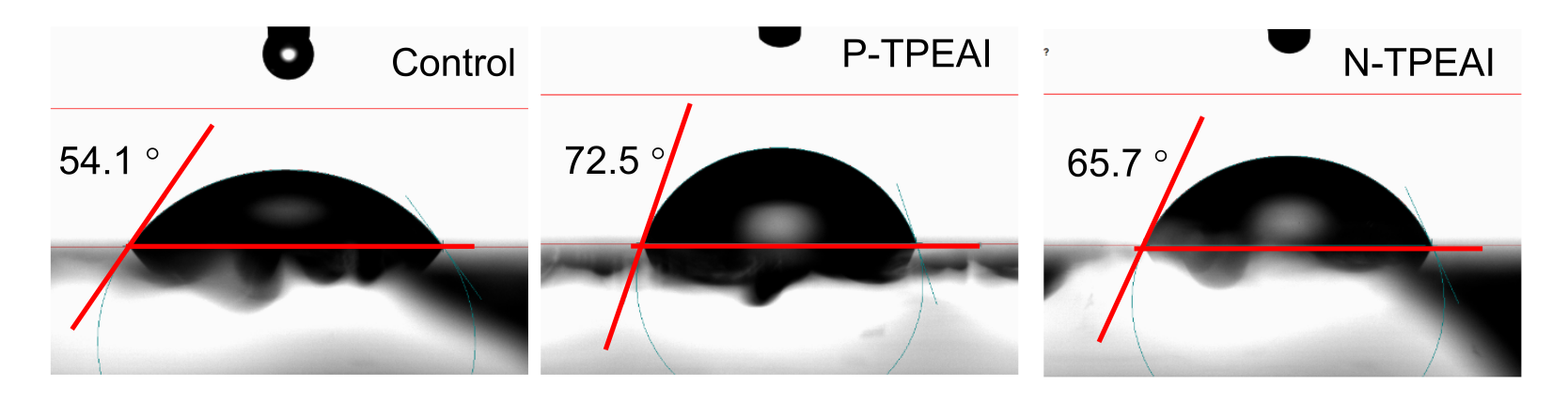


**Fig. S32** The water contact angles for control, P-TPEAI and N-TPEAI treated perovskite films.


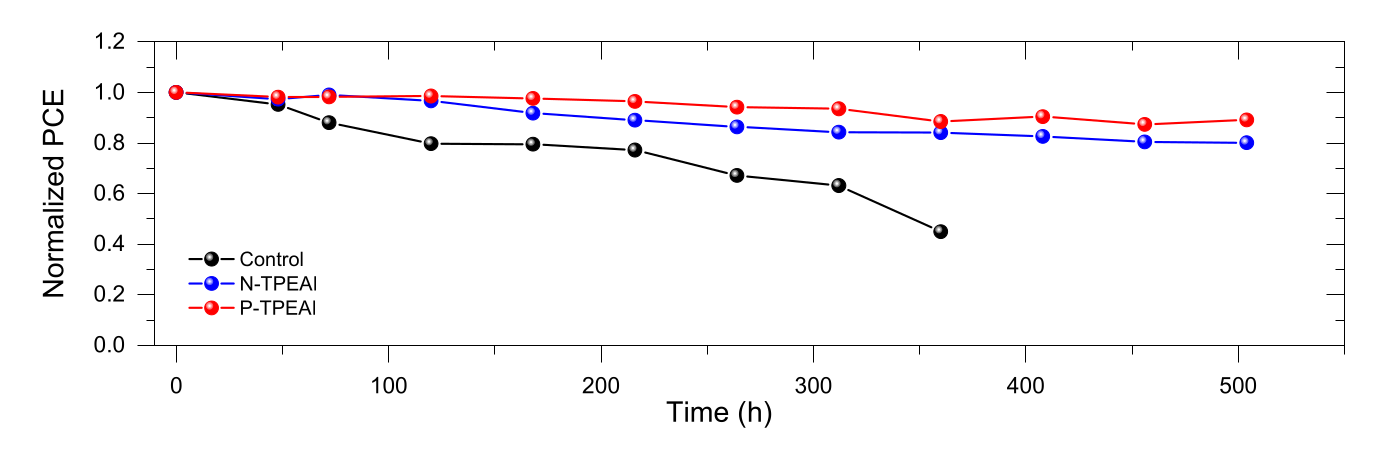


**Fig. S33** Photostability tracking under the open-circuit condition of PSCs in air atmosphere (~15°C, 25−30% RH). The initial PCE of control, N-TPEAI-treated, and P-TPEAI-treated devices are 17.8%, 21.7%, and 23.3%, respectively.

7. Supplementary Tables

**Table S1.** Parameters of simulated (P-TPEA)_2_PbI_4_ and (N-TPEA)_2_PbI_4_ 2D perovskites.

|  | (P-TPEA)_2_PbI_4_ | (N-TPEA)_2_PbI_4_ |
| --- | --- | --- |
| *a* | 6.30 Å | 6.27 Å |
| *b* | 6.17 Å | 6.24 Å |
| *c* | 29.12 Å | 27.15 Å |
| *α* | 87.07° | 85.35° |
| *β* | 92.04° | 86.59° |
| *γ* | 92.86° | 89.85° |
| Pb−I−Pb angle (axis a) | 171.42° | 163.42° |
| Pb−I−Pb angle (axis b) | 162.65° | 176.22° |
| Pb−I bond distance (axis a) | 3.15/ 3.17 Å | 3.19/ 3.17 Å |
| Pb−I bond distance (axis b) | 3.12/ 3.12 Å | 3.12/ 3.12 Å |
| Pb−I bond distance (axis c) | 3.10/ 3.30 Å | 3.20/ 3.08 Å |
| Average Pb−I bond distance (horizontal) | 3.14 Å | 3.17 Å |

**Table S2.** Parameters of N-TPEA^+^ cation pairs in simulated (N-TPEA)_2_PbI_4_ 2D perovskites.

| Naphthalene rings | NN1 | NN2 |
| --- | --- | --- |
| Angle between planes | 86.5° | 86.5° |
| Minimum C/C atom distance | 3.63 Å | 3.15 Å |
| Centroid-to-centroid distance | 5.69 Å | 6.05 Å |
| Centroid of plane 1−atoms of plane 2 | 5.13−7.36 Å | 3.99−8.47 Å |
| Centroid of plane 2−atoms of plane 1 | 3.76−8.00 Å | 5.48−6.59 Å |
| Binding energy (eV) | −15.80381 | −15.67768 |
| HOMO (Hartree) | −0.26003 | −0.26248 |
| HOMO−1 (Hartree) | −0.26972 | −0.26619 |
| Hole transfer integral (meV) | 131.8 | 50.5 |

**Table S3.** Parameters of P-TPEA^+^ cation pairs in simulated (P-TPEA)_2_PbI_4_ 2D perovskites.

| Phenyl rings | PP1 | PP2 |
| --- | --- | --- |
| Angle between planes | 27.9° | 27.9° |
| Minimum C/C atom distance | 3.06 Å | 3.18 Å |
| Centroid-to-centroid distance | 4.30 Å | 4.53 Å |
| Centroid of plane 1−atoms of plane 2 | 3.20−5.54 Å | 3.59−5.68 Å |
| Centroid of plane 2−atoms of plane 1 | 3.79−5.16 Å | 3.97−5.40 Å |
| Binding energy (eV) | −16.41694 | −16.15165 |
| HOMO (Hartree) | −0.26165 | −0.25918 |
| HOMO−1 (Hartree) | −0.27038 | −0.26717 |
| Hole transfer integral (meV) | 118.8 | 108.7 |

**Table S4.** Calculated binding energy and hole transfer integral of N-TPEA^+^/PTAA, P-TPEA^+^/PTAA and DPA-PEA^+^/PTAA.

|  | N-TPEA^+^/PTAA | P-TPEA^+^/PTAA | DPA-PEA^+^/PTAA |
| --- | --- | --- | --- |
| Binding Energy (eV) | −0.751 | −1.264 | −0.531 |
| HOMO (Hartree) | −0.06485 | −0.06117 | −0.06778 |
| HOMO−1 (Hartree) | −0.17832 | −0.17677 | −0.17788 |
| Hole Transfer Intergral (meV) | 1543.8 | 1572.8 | 1498.0 |

**Table S5.** The fitted carrier lifetime of perovskite films derived from the TRPL measurements upon 450 nm excitation.

| Perovskite | *A*_1_ | *τ*_1_ (ns) | *A*_2_ | *τ*_2_ (ns) | *τ*_avg_ (ns) |
| --- | --- | --- | --- | --- | --- |
| FAPbI_3_ | 137 | 158 | 2508 | 1760 | 1677 |
| N-TPEAI-treated FAPbI_3_ | 391 | 26.3 | 2589 | 513 | 449 |
| P-TPEAI-treated FAPbI_3_ | 466 | 7.3 | 2601 | 857 | 728 |
| FAPbI_3_/PTAA | 496 | 147 | 2221 | 868 | 736 |
| N-TPEAI-treated FAPbI_3_/PTAA | 406 | 3.2 | 2845 | 468 | 410 |
| P-TPEAI-treated FAPbI_3_/PTAA | 513 | 2.6 | 2831 | 578 | 490 |

**Table S6.** The parameters derived from the SCLC measurements based on hole-only devices.

| Perovskite | *V*_TFL_ (V) | *N*_trap_ (10^14^ cm^−3^) | *m*_h_ (10^−5^ cm^2^V^−1^s^−1^) |
| --- | --- | --- | --- |
| Control | 0.45 | 16.9 | 4.85 |
| N-TPEAI-treated | 0.22 | 8.28 | 33.7 |
| P-TPEAI-treated | 0.15 | 5.65 | 45.5 |

**Table S7.** Cyclic voltammetry and UV-vis absorption analysis of different ligands and perovskite films.

|  | *E*_on-set_ (eV) (vacuum) | HOMO (eV) | LUMO (eV) | Bandgap (eV) |
| --- | --- | --- | --- | --- |
| N-TPEAI | 0.85 eV | −5.28 | −2.14 | 3.14 |
| P-TPEAI | 0.82 eV | −5.25 | −1.86 | 3.39 |
| (N-TPEA)_2_PbI_4_ | − | − | − | 3.00 |
| (P-TPEA)_2_PbI_4_ | − | − | − | 2.74 |
| FAPbI_3_ | − | − | − | 1.54 |
| N-TPEAI treated FAPbI_3_ | − | − | − | 1.54 |
| P-TPEAI treated FAPbI_3_ | − | − | − | 1.54 |

**Table S8.** Photovoltaic parameters of PSCs with varied concentrations of N-TPEAI treatment measured under 100 mW cm^–2^ illumination (AM 1.5G).

| Devices | *V*_oc_ (V) | *J*_sc_ (mA cm^–2^) | FF (%) | PCE (%) |
| --- | --- | --- | --- | --- |
| 2 mg/ mL | 1.182 | 25.93 | 79.72 | 24.42 |
| 3 mg/ mL | 1.182 | 25.90 | 81.83 | 25.05 |
| 4 mg/ mL | 1.186 | 25.82 | 80.13 | 24.53 |

**Table S9.** Photovoltaic parameters of PSCs with varied concentrations of P-TPEAI treatment measured under 100 mW cm^–2^ illumination (AM 1.5G).

| Devices | *V*_oc_ (V) | *J*_sc_ (mA cm^–2^) | FF (%) | PCE (%) |
| --- | --- | --- | --- | --- |
| 2 mg/ mL | 1.193 | 25.86 | 81.91 | 25.26 |
| 3 mg/ mL | 1.196 | 25.94 | 83.65 | 25.95 |
| 4 mg/ mL | 1.196 | 25.93 | 81.73 | 25.34 |

**Table S10.** Photovoltaic parameters of PSCs measured at different scan directions under 100 mW∙cm^–2^ illumination (AM 1.5G).

$$HI=\frac{\mathrm{PCE}_{\mathrm{reverse}}-\mathrm{PCE}_{\mathrm{forward}}}{\mathrm{PCE}_{\mathrm{reverse}}}$$

| Devices |  | *V*_oc_  (V) | *J*_sc_  (mA cm^–2^) | FF  (%) | PCE  (%) | HI |
| --- | --- | --- | --- | --- | --- | --- |
| Control | reverse | 1.096 | 25.50 | 77.60 | 21.69 | 6.41 |
|  | forward | 1.044 | 25.50 | 76.23 | 20.30 |  |
| N-TPEAI-treated | reverse | 1.182 | 25.90 | 81.83 | 25.05 | 3.03 |
|  | forward | 1.174 | 25.83 | 80.10 | 24.29 |  |
| P-TPEAI-treated | reverse | 1.201 | 25.91 | 83.96 | 26.13 | 2.68 |
|  | forward | 1.197 | 25.85 | 82.16 | 25.43 |  |

**Table S11.** Summary of some representative photovoltaic performance of PTAA-based PSCs with PCE >22%.

|  | Device structure | *V*_oc_ (V) | *J*_sc_ (mA cm^−2^) | FF (%) | PCE (%) | Reference |
| --- | --- | --- | --- | --- | --- | --- |
| n-i-p | FTO/SnO_2_/perovskite/PYBA/PTAA/Au | 1.17 | 25.47 | 80.68 | 24.04 | [20] |
| n-i-p | FTO/TiO_2_/CNF-CsFAMA-based PSK/PTAA/Au | 1.14 | 24.00 | 82 | 22.44 | [21] |
| n-i-p | ITO/SnO_2_/Cs_0.05_FA_0.85_MA_0.10_Pb(I_0.95_Br_0.05_)_3_/PTAA (Li-BCF)/Au | 1.144 | 23.91 | 81 | 22.29 | [22] |
| n-i-p | ITO/SnO_2_/FA_0.9_MA_0.05_Cs_0.05_PbI_3_/Br4TmI/PTAA/Au | 1.13 | 25.73 | 78.96 | 22.90 | [23] |
| n-i-p | FTO/SnO_x_/SnO_2_/FA–based PSK/OAI/HFTF-PTAA/Au | 1.14 | 25.2 | 83.7 | 24.0 | [24] |
| n-i-p | ITO/SnO_2_/MA_0.10_Cs_0.05_FA_0.85_Pb(I_0.95_Br_0.05_)_3_/PTAA (Li-CYCLIC)/Au | 1.151 | 23.96 | 80.6 | 22.23 | [25] |
| n-i-p | FTO/TiO_2_/Cs_0.06_FA_0.94_PbI_3_/PTAA (PEA-TFSI)/Au | 1.05 | 25.6 | 82 | 22.1 | [26] |
| n-i-p | FTO/bl-TiO_2_/mp-TiO_2_:PSK composite layer/PSK upper layer/PTAA/Au | 1.11 | 25.0 | 81.7 | 22.6 | [27] |
| n-i-p | FTO/c-TiO_2_/perovskite/MeO-PEAI/PTAA/Au | 1.148 | 25.74 | 80.44 | 23.76 | [28] |
| n-i-p | ITO/SnO_2_/perovskite/4TeI/PTAA/Au | 1.137 | 24.9 | 83.8 | 23.7 | [29] |
| n-i-p | ITO/SnO_2_/perovskite/Cl4TmI/PTAA/Au | 1.125 | 25.96 | 84.32 | 24.63 | [4] |
| n-i-p | ITO/SnO_2_/perovskite/OAI CeOx/PTAA/Au | 1.19 | 25.4 | 82.3 | 24.93 | [30] |
| n-i-p | ITO/SnO_2_/perovskite/MeQAPyBF_4_/PTAA/Ag | 1.195 | 25.72 | 85.15 | 26.17 | [31] |
| p-i-n | ITO/PTAA/ FA_0.95_Cs_0.05_PbI_3_ (PSP)/PEAI or OAI/BAI+PDAI_2_/C_60_/ BCP/Ag | 1.164 | 26.14 | 85.74 | 26.09 | [32] |
| p-i-n | ITO/NiO_x_/PTAA/TAPT/perovskite/PCBM+C_60_/BCP/Cr/Cu | 1.159 | 26.09 | 81.2 | 24.57 | [33] |
| p-i-n | ITO/PTAA/ZrO_2_/Cs_0.05_FA_0.95_PbI_3_/C_60_/BCP/Cu | 1.192 | 25.88 | 82.9 | 25.56 | [34] |
| p-i-n | ITO/ PTAA (α-6T)/perovskite/ C_60_/BCP/Ag | 1.10 | 25.01 | 80.97 | 22.23 | [35] |
| p-i-n | ITO/PTAA/perovskite/PEAI/C_60_/BCP/Ag | 1.12 | 25.43 | 84.81 | 24.15 | [36] |
| p-i-n | ITO/PTAA/perovskite/FcTc_2_/C_60_/BCP/Ag | 1.184 | 25.68 | 82.32 | 25.0 | [37] |
| n-i-p | FTO/SnO_2_/perovskite/P-TPEAI/PTAA/Au | 1.201 | 25.91 | 83.96 | 26.13 | This work |

**Supplementary References**

[1] H. Cao, T. Li, L. Zhao, Y. Qiang, X. Zheng, et al. Triphenylamine-Based Hole-Transporting Ligands for 2D/3D FAPbI_3_ Perovskite Solar Cells. ACS Energy Lett. **10**, 2017−2025 (2025). https://doi.org/10.1021/acsenergylett.5c00471

[2] E. M. Samples, J. M. Schuck, P. B. Joshi, K. A. Willets, G. E. Dobereiner, Synthesis and Properties of N-Arylpyrrole-Functionalized Poly(1-hexene-alt-CO). Macromolecules **51**, 9323−9332 (2018). https://doi.org/10.1021/acs.macromol.8b01629

[3] J. J. Yoo, G. Seo, M. R. Chua, T. G. Park, Y. Lu, et al., Efficient perovskite solar cells via improved carrier management. Nature **590**, 587−593 (2021). https://doi.org/10.1038/s41586-021-03285-w

[4] K. Ma, J. Sun, H. R. Atapattu, B. W. Larson, H. Yang, et al., Holistic energy landscape management in 2D/3D heterojunction via molecular engineering for efficient perovskite solar cells. Science Advances **9**, eadg0032 (2023). https://doi.org/10.1126/sciadv.adg0032

[5] M. A. Green, Accuracy of analytical expressions for solar cell fill factors. Solar Cells **7**, 337−340 (1982). https://doi.org/10.1016/0379-6787(82)90057-6

[6] Z. Li, X. Sun, X. Zheng, B. Li, D. Gao, et al., Stabilized hole-selective layer for high-performance inverted p-i-n perovskite solar cells. Science **382**, 284−289 (2023). https://doi.org/10.1126/science.ade9637

[7] W. Kohn, L. J. Sham, Self-Consistent Equations Including Exchange and Correlation Effects. Phys. Rev. **140**, A1133−A1138 (1965). https://doi.org/10.1103/PhysRev.140.A1133

[8] P. E. Blöchl, Projector augmented-wave method. Phys. Rev. B **50**, 17953−17979 (1994). https://doi.org/10.1103/PhysRevB.50.17953

[9] G. Kresse, J. Furthmüller, Efficient iterative schemes for ab initio total-energy calculations using a plane-wave basis set. Phys. Rev. B **54**, 11169−11186 (1996). https://doi.org/10.1103/PhysRevB.54.11169

[10] S. Grimme, Semiempirical GGA-type density functional constructed with a long-range dispersion correction. J. Comput. Chem. **27**, 1787−1799 (2006). https://doi.org/10.1002/jcc.20495

[11] J. Hu, I. W. H. Oswald, S. J. Stuard, M. M. Nahid, N. Zhou, et al., Synthetic control over orientational degeneracy of spacer cations enhances solar cell efficiency in two-dimensional perovskites. Nat. Commun. **10**, 1276 (2019). https://doi.org/10.1038/s41467-019-08980-x

[12] M. J. Frisch, G. W. Trucks, H. B. Schlegel, G. E. Scuseria, M. A. Robb, et al., Gaussian, Inc., Gaussian 09, Revision E.01. Wallingford CT, 2013.

[13] A. D. Becke, Density-functional thermochemistry. III. The role of exact exchange. J. Chem. Phys. **98**, 5648−5652 (1993). https://doi.org/10.1063/1.464913

[14] K. Yamaguchi, Y. Takahara, T. Fueno, Ab Initio Molecular Orbital Theory; John Wiley & Sons:  New York, 1986.

[15] S. Grimme, S. Ehrlich, L. Goerigk, Effect of the damping function in dispersion corrected density functional theory. J. Comput. Chem. **32**, 1456−1465 (2011). https://doi.org/10.1002/jcc.21759

[16] S. F. Boys, F. and Bernardi, The calculation of small molecular interactions by the differences of separate total energies. Some procedures with reduced errors. Mol. Phys.**19**, 553−566 (1970). https://doi.org/10.1080/00268977000101561

[17] V. Coropceanu, J. r. m. Cornil, D. A. da Silva Filho, Y. Olivier, R. Silbey, et al., Charge Transport in Organic Semiconductors. Chem. Rev. **107**, 926−952 (2007). https://doi.org/10.1021/cr050140x

[18] M. Jeong, I. W. Choi, E. M. Go, Y. Cho, M. Kim, et al., Stable perovskite solar cells with efficiency exceeding 24.8% and 0.3-V voltage loss. Science **369**, 1615−1620 (2020). https://doi.org/10.1126/science.abb7167

[19] S.-Z. Lu, X.-Y. Li, J.-F. Liu, Molecular Orbital Analysis in Evaluation of Electron-Transfer Matrix Element by Koopmans' Theory. J. Phys. Chem. A **108**, 4125−4131 (2004). https://doi.org/10.1021/jp0380374

[20] R. Wang, X. Li, J. Qi, C. Su, J. Yang, et al., Lattice Strain Regulation Enables High-Performance Formamidinium Perovskite Photovoltaics. Adv. Mater. **35**, 2304149 (2023). https://doi.org/10.1002/adma.202304149

[21] F. Han, L. Wang, G. Cheng, L. Yang, Y. Lin., Conjugated small molecule inhibiting intrinsic ion migration and enriching electron transfer channels for stable and efficient perovskite solar cells. J. Power Sources **580**, 233364 (2023). https://doi.org/10.1016/j.jpowsour.2023.233364

[22] H. Yin, Z. Wan, J. Zhu, J. Xia, M. Azam, et al., Anionic engineering of a p-dopant enables efficient and stable perovskite solar cells. J. Mater. Chem. A **12**, 28943−28952 (2024). https://doi.org/10.1039/D4TA03732D

[23] J. Sun, S. Penukula, M. Li, M. R. Hosseinzade, Y. Tang, et al., Mechanical and Ionic Characterization for Organic Semiconductor-Incorporated Perovskites for Stable 2D/3D Heterostructure Perovskite Solar Cells. Small **20**, 2406928 (2024). https://doi.org/10.1002/smll.202406928

[24] T. Wang, Y. Zhang, W. Kong, L. Qiao, B. Peng, et al., Transporting holes stably under iodide invasion in efficient perovskite solar cells. Science **377**, 1227−1232 (2022). https://doi.org/10.1126/science.abq6235

[25] H. Zeng, F. Lin, Z. Wan, H. Yang, H. Lu, et al., Efficient and stabilized molecular doping of hole-transporting materials driven by a cyclic-anion strategy for perovskite solar cells. Chem. Sci. **15**, 9814−9822 (2024). https://doi.org/10.1039/D4SC02020K

[26] N. Nishimura, H. Kanda, R. Katoh, A. Kogo, T. N. Murakami., Thermally stable phenylethylammonium-based perovskite passivation: spontaneous passivation with phenylethylammonium bis(trifluoromethylsulfonyl)imide during deposition of PTAA for enhancing photovoltaic performance of perovskite solar cells. J. Mater. Chem. A **12**, 15631−15640 (2024). https://doi.org/10.1039/D4TA02036G

[27] W. S. Yang, B.-W. Park, E. H. Jung, N. J. Jeon, Y. C. Kim, et al., Iodide management in formamidinium-lead-halide–based perovskite layers for efficient solar cells. Science **356**, 1376−1379 (2017). https://doi.org/10.1126/science.aan2301

[28] Z. Lan, H. Huang, S. Du, Y. Lu, C. Sun, et al. Cascade Reaction in Organic Hole Transport Layer Enables Efficient Perovskite Solar Cells. Angew. Chem. Int. Edi. **63**, e202402840 (2024). https://doi.org/10.1002/anie.202402840

[29] J. Sun, K. Ma, Z.-Y. Lin, Y. Tang, D. Varadharajan, et al., Tailoring Molecular-Scale Contact at the Perovskite/Polymeric Hole-Transporting Material Interface for Efficient Solar Cells. Adv. Mater. **35**, 2300647 (2023). https://doi.org/10.1002/adma.202300647

[30] M. Lee, G. K. Asare, K. Sun, S. Yun, J. Lim, et al., Cerium Oxide Incorporation for Radiation Tolerance and Stability in Perovskite Solar Cells. ACS Energy Letters **11**, 389−400 (2026). https://doi.org/10.1021/acsenergylett.5c02116

[31] Y. Ding, L. Zhang, P. Xie, Z. Zhang, G. Qu, et al., Synergistic Bulk and Interface Passivation via Conjugated Ionic Additives Enables 26% Efficient PTAA-Based Perovskite Solar Cells. Adv. Energy Mater. **n/a**, e04647 (2025). https://doi.org/10.1002/aenm.202504647

[32] Z. Liang, Y. Zhang, H. Xu, W. Chen, B. Liu, et al., Homogenizing out-of-plane cation composition in perovskite solar cells. Nature **624**, 557−563 (2023). https://doi.org/10.1038/s41586-023-06784-0

[33] H. Chen, J. Yang, Q. Cao, T. Wang, X. Pu, et al., π-Interactions suppression of buried interface defects for efficient and stable inverted perovskite solar cells. Nano Energy **117**, 108883 (2023). https://doi.org/10.1016/j.nanoen.2023.108883

[34] B. Shao, H. Zhu, R. Zhou, L. Wang, Y. Xu, et al., Minimized Photoelectric Losses in Inverted Perovskite Solar Cells via a Discrete Photonic Scaffold. ACS Energy Letters **10**, 1030−1038 (2025). https://doi.org/10.1021/acsenergylett.4c03380

[35] J. Jiang, X. Wang, A. Li, J. Song, X.-F., Wang. Enhanced Photovoltaic Performance by Improved Hole Extraction with a Molecule Dopant in Inverted Perovskite Solar Cells. ACS Appl. Energy Mater. **7**, 3996−4003 (2024). https://doi.org/10.1021/acsaem.4c00291

[36] Y. Che, J. Deng, Y. Gao, X. Li, X. Wang, et al., Solvent-Activated Transformation of Polymer Configurations for Advancing the Interfacial Reliability of Perovskite Photovoltaics. J. Am. Chem. Soc. **146**, 26060−26070 (2024). https://doi.org/10.1021/jacs.4c05904

[37] Z. Li, B. Li, X. Wu, S. A. Sheppard, S. Zhang, et al., Organometallic-functionalized interfaces for highly efficient inverted perovskite solar cells. Science **376**, 416−420 (2022). https://doi.org/10.1126/science.abm8566
